# EAT-18 is an essential auxiliary protein interacting with the non-alpha nAChR subunit EAT-2 to form a functional receptor

**DOI:** 10.1371/journal.ppat.1008396

**Published:** 2020-04-03

**Authors:** Shivani Choudhary, Samuel K. Buxton, Sreekanth Puttachary, Saurabh Verma, Gunnar R. Mair, Ciaran J. McCoy, Barbara J. Reaves, Adrian J. Wolstenholme, Richard J. Martin, Alan P. Robertson

**Affiliations:** 1 Department of Biomedical Sciences, Iowa State University, Ames, Iowa, United States of America; 2 Department of Infectious Diseases and Center for Tropical and Emerging Global Diseases, University of Georgia, Athens, Georgia, United States of America; Imperial College London, UNITED KINGDOM

## Abstract

Nematode parasites infect approximately 1.5 billion people globally and are a significant public health concern. There is an accepted need for new, more effective anthelmintic drugs. Nicotinic acetylcholine receptors on parasite nerve and somatic muscle are targets of the cholinomimetic anthelmintics, while glutamate-gated chloride channels in the pharynx of the nematode are affected by the avermectins. Here we describe a novel nicotinic acetylcholine receptor on the nematode pharynx that is a potential new drug target. This homomeric receptor is comprised of five non-α EAT-2 subunits and is not sensitive to existing cholinomimetic anthelmintics. We found that EAT-18, a novel auxiliary subunit protein, is essential for functional expression of the receptor. EAT-18 directly interacts with the mature receptor, and different homologs alter the pharmacological properties. Thus we have described not only a novel potential drug target but also a new type of obligate auxiliary protein for nAChRs.

## Author summary

Soil-transmitted helminths affect about a quarter of the worlds’ population. Chemical anthelmintics not only alleviate the threat to human and animal health but also improve agricultural economics and food security. Here we have identified a “druggable” nicotinic acetylcholine receptor (nAChR) subunit, EAT-2, that constitutes the pharyngeal cholinergic receptor in nematodes. The receptor is required for feeding and possibly for reproductive behavior in worms. A selective therapeutic compound targeting this nAChR should either starve the worms or make them sluggish, helping with faster expulsion from the host. The EAT-2 pharyngeal nAChR is a unique receptor formed by five non-α subunits that lack vicinal cysteines in the ligand binding loop-C. To date, all cation selective nAChRs contain at least two α subunits. It is possible that EAT-2 subunits have retained functionality without the vicinal cysteines due to evolutionary modifications and expresses as a new nAChR subtype which doesn’t fit the established dogma based on the study of vertebrate receptors. Our findings also identified a new type of auxiliary protein subunit, which is essential for functional expression of the pharyngeal nAChR and also modulates its pharmacology. To the best of our knowledge, this is the first report of an auxiliary protein that is essential for functional expression in any cys-loop ligand-gated ion channel.

## Introduction

Nematodes are multicellular organisms that exhibit diverse and complex physiological behaviors. These functions are controlled by a neuromuscular system that employs a large repertoire of highly regulated transporters, neurotransmitters, peptides and ion channels, which all contribute to homeostatic cell-cell communication [1,2]. Nicotinic acetylcholine receptors (nAChRs) are pore-forming membrane proteins belonging to the cys-loop ligand-gated ion channel superfamily. They are conserved throughout metazoan evolution and characterized by a pentameric subunit organization. nAChRs facilitate rapid ionotropic neurotransmission, thereby controlling various physiological behaviors, including reproduction, navigation, feeding, and locomotion [3].

Nematode nAChRs, especially those found on somatic muscle, are targeted by the cholinergic anthelmintic drugs [3–7]. These drugs help alleviate the nematode parasite burden of the 1.5 billion people affected globally as well as mitigate the threat to global food security caused by nematode parasites of livestock [8,9]. Nematodes have a greater number (≥29) of nAChR subunits than vertebrates [10,11]; variation in stoichiometry and subunit composition leads to diverse pharmacological sensitivities which makes them attractive anthelmintic targets [6,10–12]. The introduction of recently discovered drugs such as amino-acetonitrile compounds (monepental) and spiroindoles (derquantel), which target nematode nAChRs, further highlight their importance in drug discovery [13–15].

All nAChR ion-channels are composed of five subunits forming a central ion-conducting pore and can be either homomeric (one α subunit) or heteromeric (multiple subunits with at least 2 α subunits). Nicotinic acetylcholine receptors from numerous organisms, including *Caenorhabditis elegans*, have been shown to interact with various chaperone or ancillary proteins such as RIC-3 (*r*esistance to *i*nhibitors of *c*holinesterase), UNC-50 (*unc*oordinated-50) and UNC-74 (*unc*oordinated-74). Ancillary proteins are required for correct folding, assembly of individual subunits into pentamers and trafficking of the mature nAChRs in a subtype dependent manner [12,16,17]. In addition to ancillary proteins, several structurally unrelated auxiliary subunit proteins have been identified for various ionotropic receptors [18–21]. Auxiliary proteins are essential for functional regulation of ion channels. They are non-pore forming and interact directly with the receptor subunits to modulate channel properties. They do not exhibit any channel activity on their own and are required for certain aspects of *in vivo* channel function [20]. Boulin et al. [22] identified the first auxiliary subunit for nAChRs, MOLO-1 (*mo*dulator of *l*evamis*o*le receptor-1), that regulates biological and biophysical properties of the levamisole-sensitive (L-type) nAChRs in *C*. *elegans*. This demonstrates that nAChRs are tractable to regulation by auxiliary proteins contributing to the biological and pharmacological diversity of nAChR subtypes.

In nematodes, the pharynx is a neuromuscular organ that undergoes rhythmic peristalsis to ingest food and is thus crucial for survival [23–25]. Pharyngeal peristalsis is under the control of rhythmic activation by excitatory (cholinergic) and inhibitory (glutamatergic) motor neurons innervating the pharyngeal muscle [26, 27]. Glutamate-gated chloride channels (GluCls) in the nematode pharynx are one of the primary targets for the avermectins [28–30], however little is known about the cholinergic receptors in this tissue. Through genetic screening Raizen et al. [31] identified *eat-2* (encoding a non-α nAChR subunit; *eat*ing-2) and *eat-18* (encoding a single-pass transmembrane domain protein; *eat*ing-18) as essential components of pharyngeal cholinergic transmission in *C*. *elegans*. Here, we have cloned and functionally expressed EAT-2 and EAT-18 from free-living (*C*. *elegans)* and parasitic (*Ascaris suum)* nematodes. For the first time, we find that a non-α nicotinic subunit (EAT-2) can form a homomeric ligand-gated cation selective ion-channel. The functional expression of this noncanonical receptor is dependent on co-expression with EAT-18. All previously characterized cation selective nAChRs have at least two α subunits with the ligand binding sites located at the interface between each α and its adjacent subunit [10,32]. We have used electrophysiological, biochemical, and molecular techniques to demonstrate that EAT-18 forms part of the mature receptor and functions as an obligate auxiliary protein.

## Results

### *Cel-*EAT-2 is a non-α nAChR subunit most similar to vertebrate α-7 subunits

*C*. *elegans* EAT-2 has the typical functional domains of a pentameric ligand-gated ion channel subunit: a large extracellular N-terminal domain of ~200 amino acids required for correct nAChR assembly and agonist binding; a cys-loop separated by 13 intervening amino acids; four transmembrane (TM) domains that form the ion-conducting pore; a cytoplasmic domain between TM3 and TM4 that is involved in modulation of channel activity and ion conductance; and a short extracellular C-terminus. EAT-2 is a non-α subunit as it lacks the pair of adjacent cysteine residues in loop-C required for agonist binding, still overall its sequence is most comparable to the human α-7 subunit with 55% similarity in amino acid residues (**S1 Fig**). Ligand binding occurs in a cleft formed by three loops (A, B, C) of the principal face of one α subunit and a series of beta strands from loops (D, E, F) of the complimentary interface of the adjacent subunit. All α subunits have either a YXCC or YXXCC motif in loop-C, and this motif was considered essential for ligand binding and modulating the affinity of the receptor binding site [33–35]. This lack of vicinal cysteines in the EAT-2 protein subunit suggests that the receptor channel will have different contact residues in the ligand binding pocket and a different pharmacology from other nAChRs.

### *Cel-eat-18* overlaps with the gene encoding for a CUB/LDL transmembrane protein LEV-10

*Cel-*EAT*-*18 is a small, single-pass transmembrane protein expressed in pharyngeal muscle and neurons with no vertebrate homologs [31,36]. There are two splice variants of the gene in *C*. *elegans*, encoding EAT-18c (71 aa) and EAT-18d (78 aa), which differ mainly in their C-terminal regions (**S2A Fig**). The coding sequence of EAT-18 is contained within *lev-10*, a gene encoding CUB/LDL transmembrane protein LEV-10, localized at cholinergic neuromuscular junctions. LEV-10 functions as an ancillary protein for levamisole sensitive nAChRs in *C*. *elegans* and is required for postsynaptic clustering of nAChRs in the body wall muscles. Mutants of the gene display weak levamisole resistance [37]. Interestingly, the first exon of both isoforms of *eat-18* is located in the first intron of *lev-10* (**S2B Fig**). Gally et al. [37] confirmed that *eat-18* was distinct from *lev-10* and was not involved in conferring levamisole resistance. Another distinguishing feature of EAT-18 is the intracellular N-terminal and extracellular C-terminal, which are reversed in *lev-10* (**S2C Fig**).

### *Cel-*EAT-2 forms a functional homomeric receptor when co-expressed with *Cel-*EAT-18

**Fig 1:**
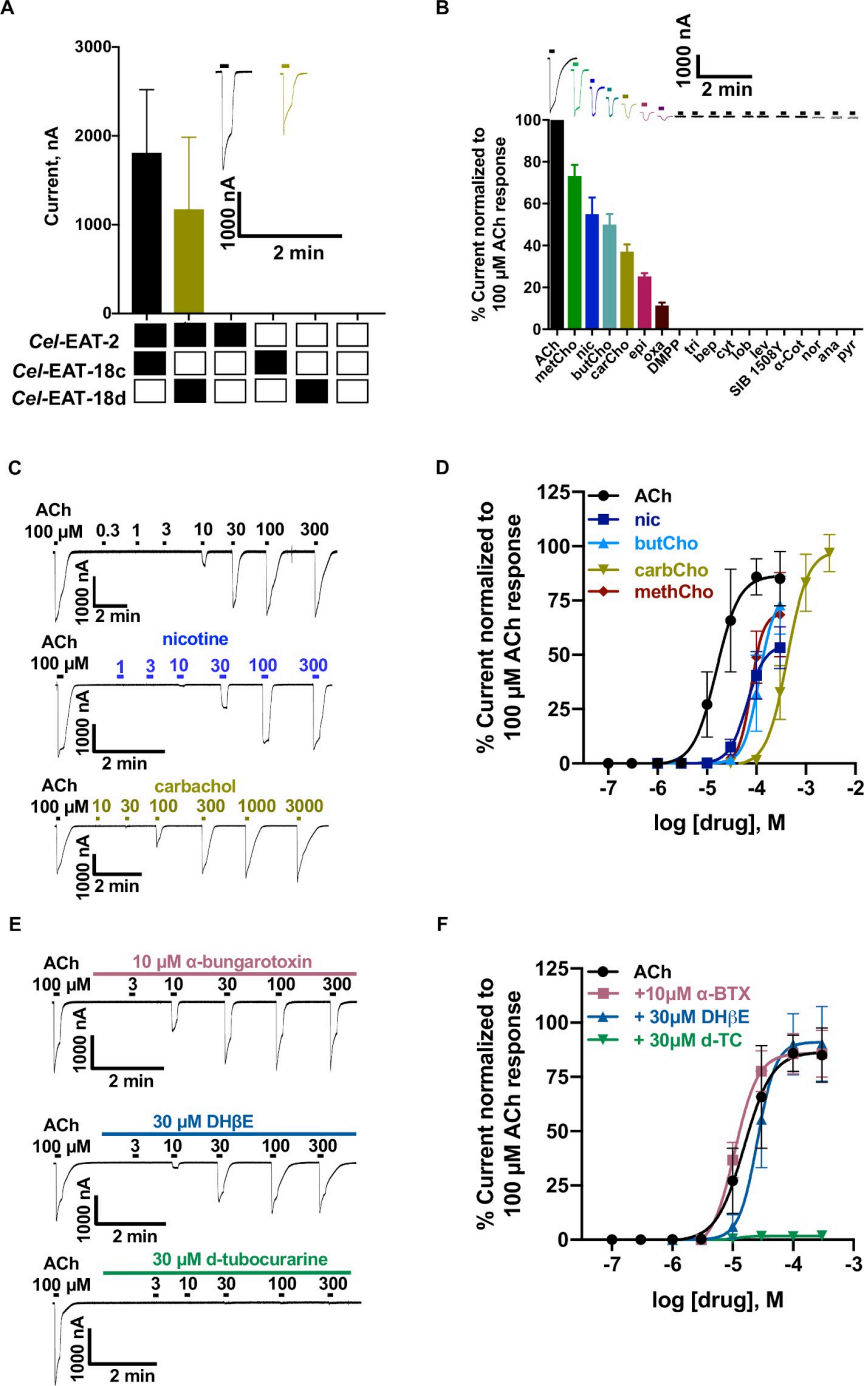
Pharmacological characterization of the *Cel-*EAT-2 nicotinic acetylcholine receptors expressed in *Xenopus* oocytes. (**A**) Current sizes (mean±S.E.M, %) produced in response to 100 μM ACh for various mixtures of *Cel-*EAT-18c & d and *Cel-*EAT-2. Black bar: *Cel-*EAT-2 with *Cel-*EAT-18c combination. Olive green bar: *Cel-*EAT-2 with *Cel-*EAT-18d combination. Black boxes indicate the presence of corresponding cRNA and empty boxes indicate the absence of cRNA in the mix. (**B**) Rank order series (expressed as mean±SEM, %, n≥6) for nAChR agonists and anthelmintics on *Cel-*EAT-2 and *Cel-*EAT-18c receptor when normalized to the control 100 μM ACh current: ACh > methacholine (methCho; 73.0±5.3) > nicotine (nic; 55.0±8.0) > butyrylcholine (butCho; 50.0±5.0) > carbachol (carCho; 37.0±3.4) > epibatidine (epi; 25.0±1.5) > oxantel (oxa; 11.0±1.3) >>> dimethylphenylpiperazine (DMPP; 0.0±0.0) = tribendimidine (tri; 0.0±0.0) = bephenium (bep; 0.0±0.0) = cytisine (cyt; 0.0±0.0) = lobeline (lob; 0.0±0.0) = levamisole (lev; 0.0±0.0) = SIB 1508Y (0.0±0.0) = 𝛼-cotinine (𝛼-cot; 0.0±0.0) = nornicotine (nor; 0.0±0.0) = anabasine (ana; 0.0±0.0) = pyrantel (pyr; 0.0±0.0). (**C**) Sample traces for ACh, nicotine and carbachol concentration–response relationships for *Cel-*EAT-2 and *Cel-*EAT-18c nAChR. (**D**) Concentration-response plots of selected agonists (n≥6) for *Cel-*EAT-2 and *Cel-*EAT-18c nAChR. *pEC*_*50*_ (mean±SEM) and Hill slope (*n*_*H*_, mean±SEM) values were respectively: 4.8±0.0 and 1.9±0.3 for ACh; 4.2±0.1 and 2.4±0.4 for nic; 4.1±0.0 and 3.5±1.3 for methCho, 3.9±0.1 and 2.8±1.8 for butCho; 3.4±0.0 and 2.1±0.3 for carbCho. (**E**) Sample traces for ACh concentration–response relationships in the presence of 10 μM *α*-bungarotoxin (*α*-BTX), 30 μM DHβE (Dihydro-β-erythroidine) and 30 μM d-tubocurarine (d-TC) for *Cel-*EAT-2 and *Cel-*EAT-18c nAChR. (**F**) ACh concentration-response curves in the presence of *α*-BTX (n = 7), DHβE (n = 6) and d-TC (n = 6) for *Cel-*EAT-2 and *Cel-*EAT-18c nAChR. d-TC caused ≈98% reduction in the mean ACh response. *α*-BTX (*pEC*_*50*_ = 5.0±0.0 and *I*_*max*_ *=* 86.0±2.4%) and DHβE (*pEC*_*50*_ = 4.6±0.0 μM and *I*_*max*_ = 91.1±4.1%) failed to show any significant antagonistic effects on the response mediated by ACh.

Initiation of the pharyngeal muscle action potential and the frequency of excitatory pharyngeal pumping are under the control of a pair of MC neurons that synapse on marginal cells in *C*. *elegans*. MC neurons release acetylcholine producing a fast depolarization of postsynaptic muscle membranes triggering an action potential. The MC neurons behave as a neurogenic pacemaker for rapid pharyngeal pumping. MC neurotransmission requires acetylcholine (ACh) and the nAChR subunit *Cel-*EAT*-2*, which is expressed in pharyngeal muscle [31,38]. In order to reconstitute the post-synaptic pharyngeal nAChR, we expressed *Cel*-EAT-2 in *Xenopus laevis* oocytes but failed to observe electrophysiological evidence for the formation of a functional nAChR. This could be attributed to the lack of vicinal cysteines in the ligand binding site required for agonist binding and pointed to a possible requirement for an additional protein or subunit. Raizen et al. [31] have shown that similar to *Cel-eat-2*, mutations in *Cel-eat-18* rendered worms incapable of MC neurotransmission and rapid pharyngeal pumping suggesting the protein may act as either an ancillary or auxiliary protein for the assembly of a functional nAChR. We co-expressed *Cel-*EAT-2 with *Cel-*EAT-18c or *Cel-*EAT-18d cRNA and recorded robust responses to 100 μM ACh in both cases (**Fig 1A**). The resulting nAChRs produced larger current amplitudes in response to ACh application when the *Cel-*EAT-18c isoform was used. All of the subsequent recordings were done using the *Cel-*EAT-18c isoform. The ability of the non-α EAT-2 subunit to express functionally as a homomeric cation selective channel when co-injected with a non-subunit protein makes this cation selective nAChR unique to date.

### Pharmacology of the *Cel-*EAT-2 nAChR

To investigate the potential of EAT-2 as a drug target, we characterized the pharmacology of the nAChR using two-electrode voltage-clamp. Different cholinergic agonists and anthelmintic agents were tested on the heterologously expressed *Cel-*EAT*-*2 receptor. All agonists were used at 100 μM, except tribendimidine, which was tested at 30 μM (n ≥ 6 for all agonists). Methacholine was the most efficacious cholinergic agonist (*I*_*max*_ = 73 ± 5.3%) followed by nicotine (*I*_*max*_ = 55 ± 8.0%). Oxantel acted as a weak agonist and produced 11 ± 1.3% of the control ACh response. However, many of the current cholinergic anthelmintic drugs such as morantel, levamisole, bephenium, tribendimidine and pyrantel did not activate the receptor. **Fig 1B** shows the rank order series for agonists and anthelmintics on the *Cel-*EAT-2 receptor when normalized to control 100 μM ACh current response: ACh > methacholine > nicotine > carbachol > butyrylcholine > epibatidine > oxantel >>> DMPP (Dimethylphenylpiperazinium) = tribendimidine = bephenium = cytisine = lobeline = levamisole = SIB 1508Y = 𝛼-cotinine = nornicotine = anabasine = pyrantel.

To further investigate the receptor pharmacology, we examined the concentration-response relationships of selected agonists (**Figs 1C, 1D** and **S3A**). 100 μM ACh was used as the internal standard for normalization. Nicotine (*pEC*_*50*_ = 4.2 ± 0.1) was the most potent agonist after ACh (*pEC*_*50*_ = 4.8 ± 0.0), whereas carbachol was least potent with a *pEC*_*50*_ = 3.4 ± 0.0. The concentration-response curves for all the agonists had Hill coefficient values greater than 1, indicating positive cooperativity, with methacholine having the steepest Hill slope (*n*_*H*_ = 3.5 ± 1.3). This suggests that the *Cel-*EAT-2 ion channel has multiple ligand binding sites consistent with other nAChRs.

To characterize the antagonist pharmacology, we tested the effects of five cholinergic antagonists on the expressed *Cel-*EAT-2 channel. The antagonists were 𝛼-bungarotoxin (10 μM), derquantel (10 μM), paraherquamide (30 μM), d-tubocurarine (30 μM) and dihydro-β-erythroidine (30 μM, DHβE). **Fig 1E** and **1F** (and **S3B Fig)** illustrate the effect of various antagonists on the ACh concentration-response relationship for *Cel-*EAT-2. d-Tubocurarine produced the most potent inhibition and almost completely blocked the response mediated by ACh (≈98% inhibition). Unlike many mammalian nAChRs, the sensitivity and efficacy of the receptor for ACh were not altered by either α-bungarotoxin or DHβE. The antagonist functional profile based on mean current (%) decrease of the control 100 μM ACh current response was: d-tubocurarine > paraherquamide > derquantel >>> 𝛼-bungarotoxin ≈ DhβE. In conclusion, the pharmacology of the *Cel-*EAT-2 receptor is distinct from previously characterized nematode and vertebrate nAChRs [6, 39–43].

### Characterization of the acetylcholine response in the *A*. *suum* pharynx

Although *C*. *elegans* is a powerful model, it is not a parasitic nematode of medical importance. In order to validate pharyngeal nicotinic acetylcholine ion channels as potential anthelmintic drug targets, it is crucial to identify and establish the presence and, in turn the pharmacology of such nAChRs in the pharynx of parasitic worms. We therefore characterized the pharmacology of the *A*. *suum* pharynx for comparison with the *Cel-*EAT-2 receptor. We employed the current-clamp technique to understand the pharmacology of the postsynaptic nAChR response. Application of 100 μM ACh on the pharyngeal preparation produced a large depolarization accompanied by an increase in membrane conductance. The ACh response was inhibited by mecamylamine, and the preparation showed negligible responses to several muscarinic agonists (**S1 Data**). This confirmed the presence of a nicotinic acetylcholine receptor in the pharynx of the parasite.

**Fig 2:**
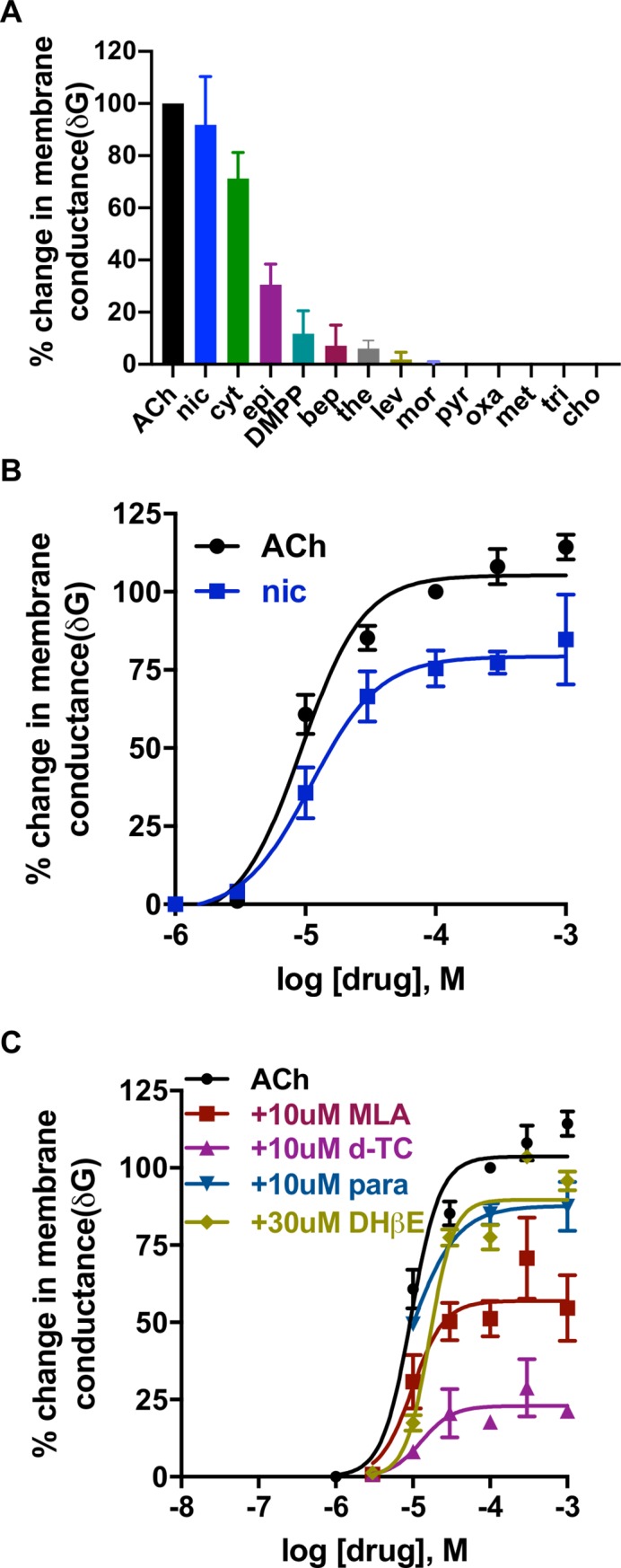
Pharmacological characterization of nAChRs expressed in the pharynx of *A*. *suum* using the current clamp technique. (**A**) Functional profile of selected vertebrate nAChR agonists and cholinergic anthelmintics producing % change in membrane conductance (δG; expressed as mean±SEM,%, n≥4): ACh (100.0±0.0) > nicotine (nic; 92.0±6.2) > cytisine (cyt; 71.0±5.0) > epibatidine (epi; 31.0±3.0) > dimethylphenylpiperazine (DMPP; 12.0±2.9) > bephenium (bep; 7.2±3.5) > thenium (the; 6.1±1.5) > levamisole (lev; 1.8±0.61) > morantel (mor; 0.3±0.3) >> choline (cho; 0.0±0.0) = pyrantel (pyr; 0.0±0.0) = oxantel (oxa; 0.0±0.0) = tribendimidine (tri; 0.0±0.0). (**B**) Concentration-conductance curves for ACh and nicotine plotting % change in conductance vs log molar concentration of the drugs. *pEC*_*50*_ (mean±SEM) and Hill slope (*n*_*H*_, mean±SEM) values were respectively: 5.0±0.0 and 1.8±0.3 for ACh (n = 6) and 5.0±0.1 and 1.7±0.6 for nicotine (n = 8). (**C**) Concentration-conductance plots of ACh in the presence of nAChR antagonists: paraherquamide (para; 10μM), methyllycaconitine (MLA; 10μM), d-tubocurarine (d-TC; 10μM) and Dihydro-β-erythroidine (DHβE; 30μM). The *pEC*_*50*_ values were 5.0±0.1 in the presence of MLA (n = 8); 4.9±0.2 in the presence of d-TC (n = 3); 5.1±0.1 in the presence of para (n = 3) and 4.8±0.0 in the presence of DHβE (n = 7). The maximal response (δG) (mean±SE, μS) values were: 57.0±4.6 in the presence of MLA; 23.0±3.0 in the presence of d-TC; 87.7±5.7 in the presence of para; 89.7±3.0 in the presence of DHβE.

We next quantified the effects of selected nicotinic agonists to determine whether the pharyngeal nAChRs are pharmacologically distinct from those of somatic muscle. Our pharyngeal preparations in this group had a mean resting membrane potential of -21.3 ± 1.3 mV and a mean resting conductance (G) of 136.4 ± 14.9 μS (n = 17). The change in conductance (δG) responses to test applications of selected nicotinic agonists were normalized to the ACh δG. Nicotine was the most potent agonist after ACh with mean δG of 92.0 ± 6.2%. Cytisine also produced a large conductance change in the *A*. *suum* pharynx (mean δG = 71.2 ± 5.0). The rank order series for vertebrate nicotinic agonists on the *A*. *suum* pharynx was: ACh > nicotine > cytisine > epibatidine > DMPP >> choline (**Fig 2A and S4A Fig**). The rank order series of selected vertebrate nicotinic agonists on the pharynx is different from that of somatic muscle nAChRs and vertebrate host nAChRs (**S1 Table**). We also tested nine cholinergic anthelmintics on the pharynx to study their effect. Our pharyngeal preparations in these experiments had a mean resting membrane potential of -19.3 ± 1.1 mV and a mean resting conductance of 150.5 ± 11.9 μS (n = 21). The δG responses to test applications of selected cholinergic anthelmintic agents were normalized to the ACh δG. **Fig 2A** (**S4A Fig**) shows the rank order series on the *A*. *suum* pharynx: ACh >> bephenium > thenium > levamisole ≈ morantel ≈ pyrantel ≈ oxantel ≈ tribendimidine. In contrast to somatic muscle nAChRs, none of the cholinergic anthelmintics tested on the pharynx produced >7% of the ACh response (**S1 Table**).

To further investigate the receptor, we used selected nicotinic antagonists (30μM) to study their inhibitory effects on 100μM ACh responses. Our pharyngeal preparations in this group had a mean resting membrane potential of -20.2 ± 1.1 mV and a mean resting conductance of 129.2 ± 6.8 μS (n = 34). The δG produced by a control application of ACh was set as 100%. We calculated the % inhibition of the δG response to ACh by nicotinic antagonists to determine a rank order series (mean ± SEM, **S5 Fig and S6 Fig**): d-tubocurarine > mecamylamine > methyllycaconitine > paraherquamide > derquantel > hexamethonium > DHβE. The functional spectrum of nicotinic receptor antagonists on the pharynx is distinct from that of vertebrate nAChRs (**S1 Table**).

We also determined concentration-response curves by plotting the concentration of agonists (1-1000μM, applied for 10s) against the response normalized to 100 μM ACh (applied for 10s) δG within each experiment. **Fig 2B** (**S4B Fig** and **S4C Fig**) shows the concentration-response curves for ACh and nicotine. The *pEC*_*50*_ of ACh and nicotine were 5.0 ± 0.0 and 5.0 ± 0.1, respectively. The maximal response of ACh was 105.3 ± 2.5 μS, and nicotine was 79.3 ± 4.3 μS. We determined the ACh concentration-response relationships in the presence of nicotinic receptor antagonists: d-tubocurarine (10μM), methyllycaconitine (10μM), paraherquamide (10μM) and dihydro-β-erythroidine (30μM) (**Fig 2C**). The *pEC*_*50*_ of the ACh concentration-response curve did not significantly differ in the presence of methyllycaconitine, d-tubocurarine or paraherquamide but the maximal response for ACh was inhibited. This suggests that these compounds act as non-competitive antagonists of the pharyngeal ACh response.

### Functional expression of *Asu*-EAT-2 requires *Asu-*EAT-18 and *Asu-*RIC-3

The pharmacological characterization of *A*. *suum* pharyngeal nAChRs revealed significant differences from the *Cel-*EAT-2 ion channel. In particular, cytisine which produced large depolarization in the *A*. *suum* pharynx, failed to activate *Cel-*EAT-2 ion channel. These pharmacological differences encouraged us to identify the subunits which constituted pharyngeal nAChRs in *A*. *suum*. We used *Cel*-EAT-2 and *Cel*-EAT-18 sequences as queries in BLASTP homology searches and identified homologs for EAT-2 and EAT-18 in the pig parasite. Comparison of *Asu-*EAT-2 with *Cel-*EAT-2 sequences revealed 80% similarity in amino acid composition, with differences among some of the ligand binding residues from various loops (**S1 Fig**). This suggested that the receptor channel could have different contact residues in the ligand binding pocket and possibly a different pharmacology. The proteins were expressed *in vitro* in *Xenopus* oocytes to recapitulate the pharyngeal ligand-gated cation channel. Unlike *Cel-*EAT-2 nAChRs, *Asu-*EAT-2 not only required *Asu-*EAT-18 but also *Asu-*RIC-3 for robust expression. However, the addition of *Asu-*UNC-50 and *Asu-*UNC-74 (*I*_*max*_ ± SEM = 37.0 nA ± 5.2 nA) did not produce any significant increase in current amplitude. The most robust responses (*I*_*max*_ ± SEM = 418.0 nA ± 40.8 nA) were observed from oocytes injected with 30 ng each of *Asu-*EAT-2 and *Asu-*EAT-18 plus 20 ng of *Asu-*RIC-3 (**S7A Fig**), and so these cRNA amounts were used for all subsequent injections.

### Pharmacological profile of the *Asu-*EAT-2 nAChR

**Fig 3:**
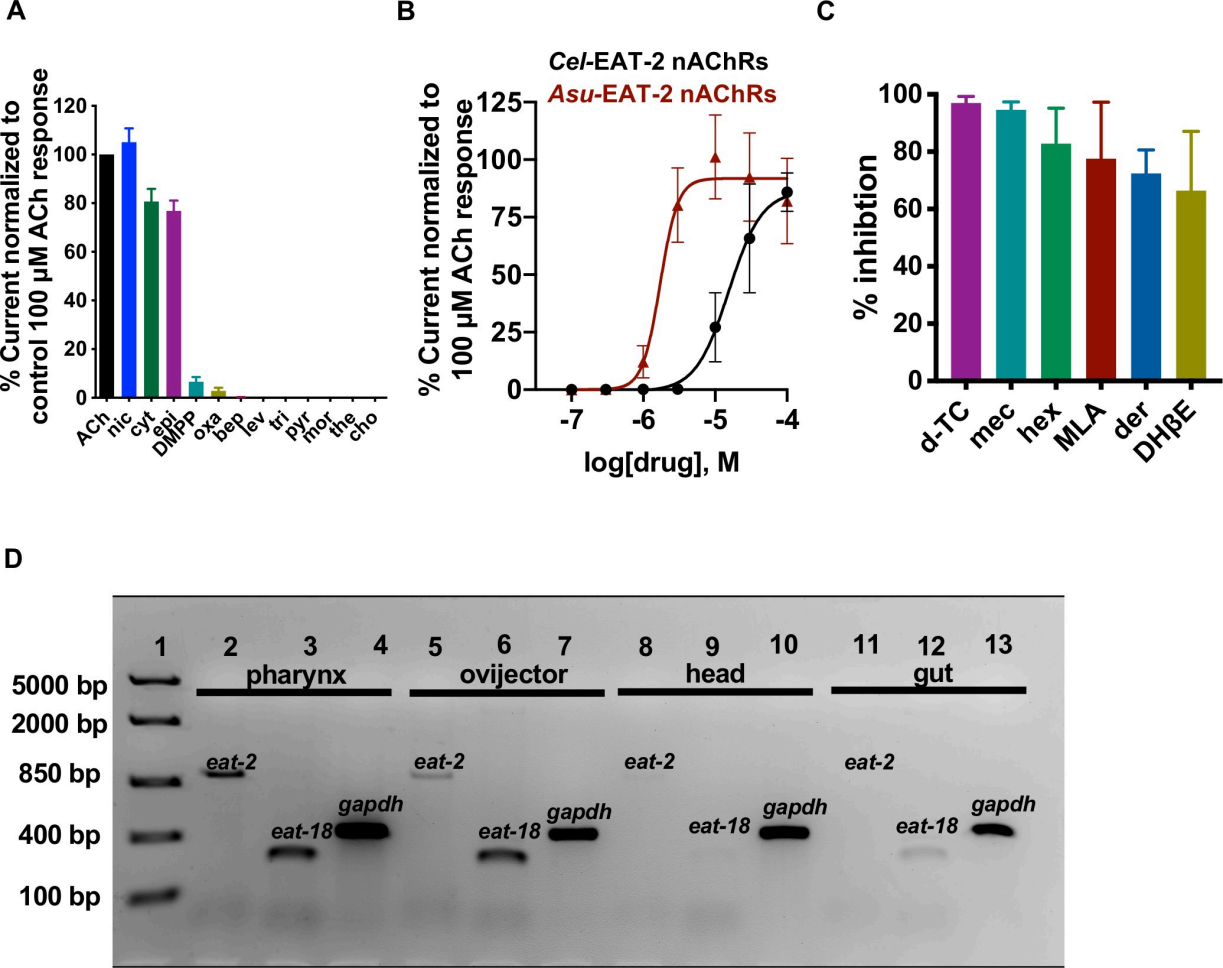
Effect of selected cholinergic agonists, anthelmintics and antagonists on the *Asu-*EAT-2 receptor expressed in *Xenopus* oocytes. (**A**) Functional profile (mean ± SEM, %, n≥5) of cholinergic agonists and anthelmintics when normalized to the control 100 μM ACh current: nicotine (nic; 105.0±5.7) ≈ ACh (100±0.0, n = 9) > cytisine (cyt; 81.0±5.2) > epibatidine (epi; 77.0±4.2) > dimethylphenylpiperazinium (DMPP; 6.6±1.9) > oxantel (oxa; 3.0±1.3) >>> bephenium (bep; 0.1±0.1, n = 9) > levamisole (lev; 0.0±0.0) = tribendimidine (tri; 0.0±0.0) = pyrantel (pyr; 0.0±0.0) = morantel (mor) = thenium (the; 0.0±0.0) = choline (cho; 0.0±0.0). (**B**) Comparison of concentration-response plots to ACh for the *Cel*-EAT-2 (black curve) and *Asu*-EAT-2 (maroon curve) receptor. *pEC*_*50*_ (mean ± SEM) and Hill slope (*n*_*H*_, mean ± SEM) values were respectively: 4.8 ± 0.0 and 1.9 ± 0.2 for *Cel*-EAT-2 (n = 9); 5.8 ± 0.1 and 3.5 ± 1.1 for *Asu*-EAT-2 (n = 6). (**C**) Functional profile (expressed as mean ± SEM, %, n = 6) of selected vertebrate nAChR antagonists (30 μM) based on inhibition of ACh (100 μM) mediated currents. d-Tubocurarine (d-TC; 97±1.0) and mecamylamine (mec; 95±1.1) almost completely blocked the ACh response. Mean current inhibition were 83.0±5.1 for hexamethonium (hex), 78.0±8.1 for methyllycaconitine (MLA), 72.0±3.4 for derquantel and 66±8.4 for DHβE (Dihydro-β-erythroidine). (**D**) Localization of *Asu-eat-2* and *Asu-eat-18* mRNA in different body tissues of the *A*. *suum* worm (n = 5). RT-PCR analysis of *Asu-eat-2* (lanes 2, 5, 8, 11) and *Asu-eat-18* (lanes 3, 6, 9, 12) and *gapdh* control (lanes 4, 7, 10, 13) in pharynx, ovijector, head, and gut region. The PCR product sizes for *eat-2*, *eat-18* and *gapdh* were 949, 213 and 411 bp respectively. Lane 1, FastRuler High Range DNA ladder.

We were interested in determining the comparative pharmacological profile of *A*. *suum* pharyngeal nAChRs and *Asu*-EAT-2 ion channel in order to establish the contribution of the non-α subunit in pharyngeal pharmacology. We used similar cholinergic agonists, anthelmintic agents, and antagonists as *in vivo A*. *suum* pharyngeal experiments on the expressed *Asu-*EAT-2 receptor. The rank order series of cholinergic agonists and anthelmintic agents based on maximum current response (**Fig 3A and S7B Fig**) for the receptor was: nicotine > ACh > cytisine > epibatidine > DMPP > oxantel. As with the *A*. *suum* pharynx, cholinomimetic anthelmintics such as bephenium, tribendimidine, levamisole, and pyrantel failed to activate the receptor. We also constructed a concentration-response curve for ACh and found it to be ≈ 9 times more potent on the *Asu*-EAT-2 nAChR compared to *Cel*-EAT-2 with a *pEC*_*50*_ = 5.7 ± 0.0 (**Fig 3B and S7C Fig**).

We tested the antagonistic effects of derquantel, mecamylamine, d-tubocurarine, DHβE, hexamethonium, and methyllycaconitine on the *Asu-*EAT-2 receptor. The mean % inhibition of the 100 μM ACh current response was used to determine the effect of the antagonists. Mecamylamine and d-tubocurarine produced almost 100% inhibition of the ACh currents, and DhβE was the least potent antagonist (inhibition, 66 ± 8.4%). The functional profile for the antagonists (**Fig 3C and S7D Fig**) was: d-tubocurarine ~ mecamylamine > hexamethonium > methyllycaconitine > deraquantel > DhβE. The rank order series of cholinomimetic anthelmintics, nicotinic agonists, and antagonists on the *Asu-*EAT-2 receptor differs from that of the *A*. *suum* somatic muscle nAChRs as well as the vertebrate nAChRs (**S1 Table**). In conclusion, the *Asu-*EAT-2 receptor has a distinct pharmacology and is, therefore, likely suitable to be exploited as a therapeutic target.

### Tissue expression of *eat-2* and *eat-18* in *A*. *suum*

In *C*. *elegans*, EAT-2 expression is restricted to pharyngeal muscle, while EAT-18 is found in both pharyngeal muscle and some neurons [36]. We used RT-PCR to examine the distribution of *Asu-eat-2* and *Asu-eat-18* mRNA in various dissected adult *A*. *suum* tissues and single somatic muscle cells (n≥ 5; **Fig 3D**). We determined that *Asu-eat-2* was transcribed in the pharynx, sections of the reproductive tract, and the head region. RT-PCR results revealed the presence of *Asu-eat-18* message in the same tissues, as well as gut tissue. We found no evidence of expression of *Asu-eat-2* or *Asu-eat-18* in somatic muscle cells (**S8 Fig**). The widespread expression of both the proteins in body tissues other than pharynx was unexpected. It is plausible that *Asu-*EAT-2 not only assists in feeding but also plays a role in other physiological processes such as reproduction. It also raises the possibility that EAT-18 is interacting with other nAChR subunits in different tissues.

### Comparative pharmacological profile reveals EAT-2 constitutes the pharyngeal nAChR in *A*. *suum*

**Fig 4:**
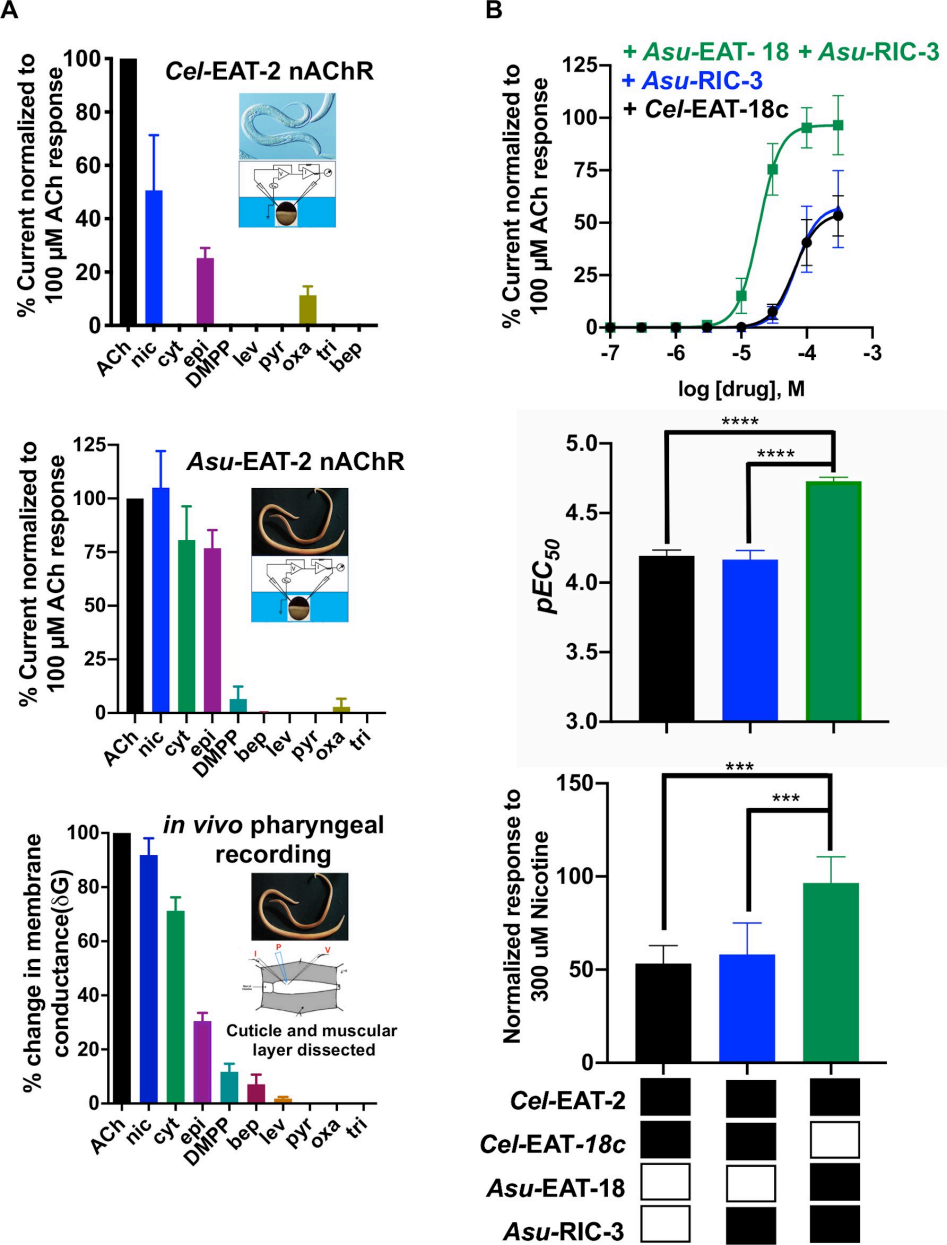
Comparative pharmacological profile of agonists on *in vivo* and *in vitro* pharyngeal receptors. (**A**) Comparative pharmacology of agonists for *Cel-*EAT-2 receptor expressed *in vitro*, *Asu-*EAT-2 receptor expressed *in vitro*, and *in vivo* pharyngeal recording in *A*. *suum*. Inset: Images of source nematode (*C*. *elegans* and *A*. *suum*) and corresponding recording techniques (TEVC recordings from *X*. *laevis* oocytes and current-clamp recordings from intact *A*. *suum* pharynx). (**B**) Effect of different EAT-18 homologs on the pharmacology of the *Cel-*EAT-2 receptor. Concentration-response curves showing comparison for nicotine application on *Cel-*EAT-2 + *Cel-*EAT-18c mix (black curve), *Cel-*EAT-2 + *Cel-*EAT-18c + *Asu-*RIC-3 (blue curve) and *Cel-*EAT-2 + *Asu-*EAT-18 + *Asu-*RIC-3 mix (green curve). Bar graphs showing a significant effect of using different EAT-18 proteins with *Cel-eat-2* on *pEC*_*50*_ (top graph) and on the maximum response (bottom graph) produced by application of 300 μM nicotine. ^***^P < 0.001, ^****^P < 0.0001; significantly different as indicated; Tukey’s multiple comparison tests.

**Fig 4A** shows the pharmacological comparison between *in vitro Cel-*EAT-2, *Asu-*EAT-2, and *in vivo A*. *suum* pharyngeal recordings. The agonist rank order series acquired from both *in vivo* and *in vitro* recordings in *A*. *suum* revealed a similar pharmacological profile. Both nicotine and cytisine were highly efficacious in *in vivo* and *in vitro* recordings in *A*. *suum*, while DMPP acted as a weak agonist. In comparison, the *Cel-*EAT-2 channel failed to respond to cytisine and DMPP application but was activated by oxantel (*I*_*max*_ = 11.0 ± 1.3% of ACh response) albeit weakly. Importantly, the comparable pharmacological profile observed for *Ascaris in vivo* and *in vitro* recordings suggests it is likely that EAT-2 and EAT-18 constitute the pharyngeal nicotinic response in the parasitic nematode.

### Different EAT-18 homologs affect the pharmacology of the EAT-2 nAChR

We expected to see differences in nAChR pharmacology between *A*. *suum* and *C*. *elegans* due to differences in the amino acid residues of the EAT-2 protein sequences. EAT-2 cannot form a functional receptor on its own and requires EAT-18. To determine the pharmacological relevance of EAT-18, we expressed *Cel-*EAT-2 with *Asu*-EAT-18. We tested five agonists on the expressed channel: ACh, nicotine, cytisine, levamisole, tribendimidine, and pyrantel (**S9A Fig**). No significant differences were observed in the rank order series. Interestingly, the sensitivity of nicotine was affected, illustrating a change in the pharmacology (**Fig 4B**). Substitution of *Asu*-EAT-18 for *Cel*-EAT-18c shifted the concentration-response curve to the left and increased the efficacy of nicotine on the receptor. The *EC*_*50*_ = 18.7 μM (*pEC*_*50*_ = 4.7 ± 0.0) for nicotine was approximately 3.5 times lower than before 64.2 μM (*pEC*_*50*_ = 4.2 ± 0.0). We also observed a significant increase in *I*_*max*_ (96.5 ± 2.3% from 54.6 ± 2.6%) when replacing *Cel-*EAT-18c with *Asu*-EAT-18. The sensitivity of the pharyngeal receptor to ACh was also altered significantly, but there was no effect on the agonist efficacy (**S9B Fig** and **S9C Fig**). The significant shift in ACh and nicotine *pEC*_*50*_ establishes the electrophysiological evidence of modulation of the receptor by EAT-18 and points to an interaction between the proteins.

### EAT-18 co-localizes with EAT-2 on the oocyte surface membrane

**Fig 5:**
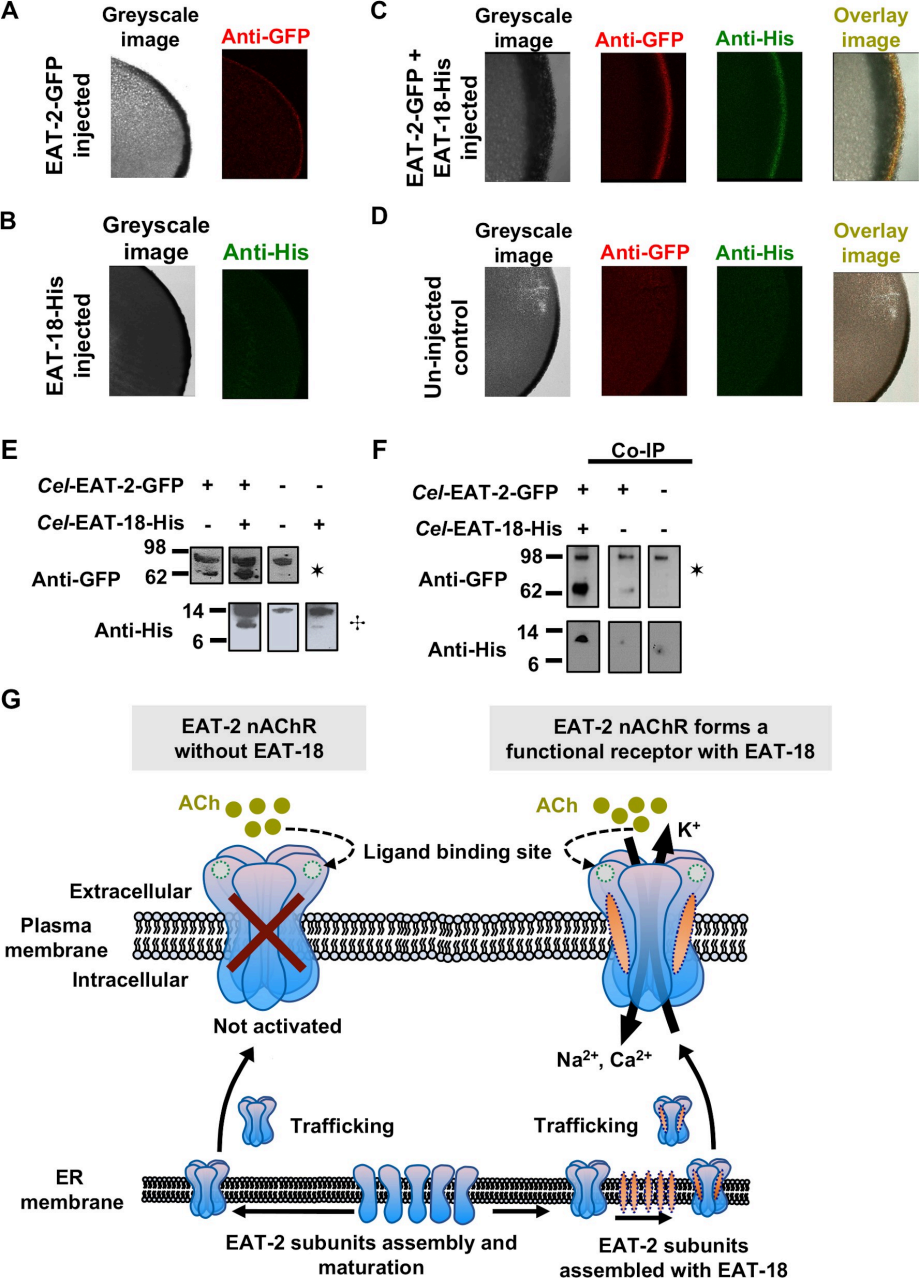
EAT-2 and EAT-18 form a receptor complex. (**A**) Immunostained oocyte sections showing expression of *Cel-*EAT-2-GFP (red fluorescence; n = 4) on the surface membrane when injected alone. (**B**) *Cel-*EAT-18-His (n = 4) fails to localize on the surface membrane when injected alone. (**C**) Double immunostained sections of *Xenopus laevis* oocytes showing *Cel-*EAT-2-GFP and *Cel-*EAT-18-His (n = 6) on the surface membrane. The overlay image (yellow fluorescence) shows the co-localization of both the proteins. (**D**) Double immunostained sections of un-injected (negative control; n = 6) *Xenopus laevis* oocytes. (**E**) Western blot analysis of *Xenopus* oocyte extracts. Un-injected oocytes served as a negative control. *Cel-*EAT-2-GFP was immunostained with anti-GFP antibodies and was present in the extracts prepared from oocytes co-injected with *Cel-*EAT-18-His as well as oocytes injected with *Cel-*EAT-2-GFP alone. *Cel-*EAT-18-His was immunostained with anti-His antibodies and was present in the extracts prepared from oocytes co-injected with *Cel-*EAT-2-GFP. ✶, ✣: non-specific interacting protein bands labeled by anti-GFP and anti-his antibodies, respectively, served as a positive control. (**F**) Co-immunoprecipitation experiments revealed *Cel-*EAT-18 directly interacts with EAT-2 and constitute part of the receptor complex. *Cel-*EAT-2-GFP was immunoprecipitated using anti-GFP, followed by Western blot analysis of *Cel-*EAT-18-His using anti-His antibodies. Un-injected oocytes and oocytes injected with *Cel-*EAT-2-GFP alone served as negative controls for co-immunoprecipitation experiments. ✶: non-specific interacting protein bands labeled by anti-GFP and anti-his antibodies, respectively, served as a positive control. (**G**) Schematic representation for physical interaction between EAT-2 and EAT-18.

EAT-18 is required for functional *in vitro* expression of EAT-2 and modulates its pharmacological properties. McKay et al. [36] have shown that *Cel*-EAT-18 is not required for the trafficking of *Cel*-EAT-2 to the oocyte membrane. It is possible that *Cel*-EAT-18 functions as an auxiliary subunit rather than an ancillary/chaperone protein. To test this hypothesis, we performed confocal imaging experiments on oocytes expressing GFP tagged *Cel-*EAT-2 and His tagged *Cel-*EAT-18c constructs. These experiments revealed that both *Cel-*EAT-2 and *Cel-*EAT-18c were co-localized on the oocyte surface membrane. **Fig 5C and 5D** show the images for double immunostained sections of injected and un-injected oocytes. *Cel*-EAT-18c is not a typical nAChR subunit protein, and its co-localization with *Cel-*EAT-2 suggests the possibility of an association between both proteins. *Cel-*EAT-2-GFP, when expressed alone, localizes to the oocyte surface membrane suggesting that *Cel-*EAT-18c does not function as an ancillary protein (**Fig 5A**).

We further assessed the expression of the *Cel-*EAT-2 channel by Western blot analysis of oocyte protein extracts. Using antibodies that recognize GFP and His tags, we detected *Cel-*EAT-2-GFP as a 62 kDa and *Cel-*EAT-18c-His as a 10 kDa protein (**Fig 5E** and **S10A Fig**). We were able to detect *Cel-*EAT-2 in membrane extracts prepared from oocytes co-injected with *Cel-*EAT-2 and *Cel-*EAT-18c as well as oocytes injected with *Cel-*EAT-2 alone. In contrast, *Cel-*EAT-18c was only present in membrane extracts prepared from oocytes co-injected with both *Cel-*EAT-2 and *Cel-*EAT-18c. We did detect *Cel-*EAT-18c in whole oocyte extracts when *Cel-*EAT-18c was injected alone. It is plausible that EAT-18 requires EAT-2 for trafficking to the surface membrane, and its role is more complicated than a simple ancillary protein; perhaps related to the functionality of the mature receptor.

### EAT-18 forms a part of the EAT-2 nAChR complex

Although *Cel-*EAT-18c was co-localized with *Cel-*EAT-2 on the surface membrane of the oocytes and modulated the pharmacology of pharyngeal nAChR, it did not prove the molecular interaction. Therefore, we performed co-immunoprecipitation experiments to explore a possible direct interaction between *Cel*-EAT-18c and *Cel*-EAT-2 (**Fig 5F** and **S10B Fig**). We were able to demonstrate that *Cel*-EAT-18c-His co-immunoprecipitated with *Cel*-EAT-2-GFP, which shows that EAT-18 directly interacts with EAT-2 and is a part of the mature receptor complex (**Fig 5G**).

## Discussion

nAChRs are vital components of the metazoan neuromuscular junction and essential targets for anti-parasitic interventions. They are typically composed of 5 subunits, including at least 2 α subunits. Here we describe for the first time a non-α nAChR subunit that can form a functional homomeric cation selective receptor when coexpressed with *Cel*-EAT-18. Even though EAT-2 lacks the essential vicinal cysteines in loop-C, the pharyngeal subunit contains most of the residues that form the “aromatic box” of the α-7 ligand binding site [44]. These include a W149 (loop-B; when mature peptide numbering of α7 subunits is used) and Y197 (loop-C) contributed by the “principle face” of one subunit and W54 (loop-D) provided by the “complimentary” face of the adjacent subunit (**S1 Fig)**. This could explain how a non-α subunit, EAT-2, was able to constitute a functional cation selective receptor. Acetylcholine-gated chloride channels are another example of cys-loop ligand-gated subunits that lack the vicinal cysteines but still form functional channels, albeit anion selective [45]. It is possible that through evolution, nematodes express additional nicotinic acetylcholine receptors subtypes in addition to the relatively small but widely studied group of receptors from vertebrates. Variable residues neighboring the conserved core of the aromatic residues and the non-conserved loop-E and Loop-F account for the differences in pharmacological properties of nicotinic acetylcholine receptors [32]. The differences in the pharmacological profile of vertebrate receptors and somatic nematode nAChRs could be attributed to these variations in amino acids.

*Cel*-EAT-18 functions as an obligate auxiliary protein and modifies the pharmacological properties of this cys-loop ion channel. Several previously characterized nAChRs require ancillary proteins, either RIC-3 alone or in combination with UNC-50 and UNC-74, for successful *in vitro* expression [6,12,16,17,39]. *In vitro* expression of *Cel*-EAT-2 receptors instead only required co-expression with EAT-18, which has little similarity to ancillary proteins. Both proteins are localized in the pharyngeal muscles, and mutations in *Cel*-*eat-18* caused pharyngeal pumping defects similar to *Cel*-*eat-2* mutations [31,36]. Based on our confocal imaging studies, *Cel-*EAT-18c is localized on the oocyte surface only when injected with *Cel-*EAT-2. This supports our hypothesis that EAT-18 interacts with EAT-2 and likely requires the pore-forming subunit protein for trafficking to the surface membrane. Other evidence of interaction is provided by the pharmacological modulation of the ACh and nicotine responses by using EAT-18 proteins from different nematode species with *Cel*-EAT-2. The co-immunoprecipitation experiments have provided more concrete evidence of direct physical interaction between *Cel-*EAT-2 and *Cel-*EAT-18. EAT-18 modifies the pharmacological properties of the EAT-2 nAChR and is necessary for *in vivo* cholinergic MC neurotransmission, which regulates pharyngeal pumping. Therefore EAT-18 meets the criteria of an auxiliary protein. However, unlike other auxiliary proteins, successful expression of a functioning channel requires the presence of EAT-18. Various auxiliary subunits have been discovered for the ionotropic glutamate-gated (kainate and AMPA) and GABA_B_ receptors that modulate their properties or functional expression levels [18–21]. The interaction of these auxiliary subunits with the receptor complex holds a physiological relevance. MOLO-1 (modulator of levamisole receptor-1) is the first example of an auxiliary subunit for levamisole sensitive acetylcholine receptors in *C*. *elegans* [22]. Like MOLO-1, null mutants of EAT-18 result in significant physiological defects, and many of the nematode species express highly conserved orthologues of EAT-18 (**S12 Fig**) suggesting evolutionary conservation of function. In contrast to EAT-18, MOLO-1 is not required for the functional expression of the somatic levamisole nAChR but only regulates the trafficking, localization, or gating kinetics; instead, EAT-18 is essential for EAT-2 to form a functional receptor *in vitro*. This implies that EAT-18 not only meets the criteria for an auxiliary protein but may belong to a novel class of proteins not previously described, adding to the types of auxiliary subunits identified for the cys-loop cation channels.

Identification of a suitable target and its validation is one of the most important steps in developing a new drug. An ideal anthelmintic target should meet certain criteria in order to be considered relevant for pharmacological intervention; important physiological function, conservation across parasite species and pharmacological divergence from host receptors. Parasite nAChRs are regarded as popular targets because they contribute to vital physiological functions. Additionally, their diversity, conserved structure among various species of nematodes, and distinct pharmacology from mammalian orthologues make them “druggable”. The pharynx is a muscular organ required for feeding in nematodes. While the nematode pharynx has been exploited as a target tissue for the avermectins (GluCls) [26,27,30,46], less is known about the nAChRs in this tissue. In *C*. *elegans* two genes, *eat-2* and *eat-18*, were required for MC neurotransmission. *Cel-*EAT-2 and *Cel-*EAT-18 are both localized in the pharyngeal muscles, and mutations in these genes caused defects in feeding behavior in the worms [31,36]. We hypothesize that activation of the pharyngeal nAChR formed by the EAT-2 subunit and EAT-18 auxiliary protein will lead to an effect similar to levamisole in somatic muscle and cause pharyngeal paralysis in nematodes. We were able to successfully co-express EAT-2 and EAT-18 from *C*. *elegans*, a model nematode, and *A*. suum, a parasitic species, in *Xenopus* oocytes and characterize the pharmacology of this conserved receptor.

### The pharyngeal nAChR composed of EAT-2 and EAT-18 as a novel drug target

The pharyngeal cys-loop ligand-gated ion channel formed by EAT-2 meets the criteria for a suitable anthelmintic drug target [47]: 1) it performs a neuromuscular function essential for parasite biology; 2) this receptor is druggable, it has distinct pharmacology from somatic muscle receptors and is insensitive to many of the currently used cholinergic anthelmintics including morantel, tribendimidine, and pyrantel; 3) EAT-2 and EAT-18 are present in multiple relevant parasitic nematode species (**S11 Fig and S12 Fig**) and the protein sequences are highly conserved; 4) *Cel-*EAT-2 is only 36% identical to human α-7 nAChR subunit, and there are no mammalian homologs for EAT-18 providing potential for selectivity; it is also pharmacologically distinct from vertebrate nAChRs. In order to identify novel drug targets for anthelmintic agents, it is crucial to understand the properties and function of the target proteins. We have successfully elucidated the components and pharmacological profile of the pharyngeal nAChR by employing various molecular, biochemical, and electrophysiology techniques.

## Methods

### Molecular biology

Plasmid constructs (Life Technologies Inc., USA) containing *C*. *elegans* EAT-2 (Accession number: Y48B6.4) & EAT-18 (Accession number: isoform-c—Y105E8A.7c.1 and isoform-d -Y105E8A.7d.1) were cloned into *XhoI* and *ApaI* restriction sites of the pTB-207 expression vector using In-Fusion cloning kit (Takara Bio USA, Inc.; EAT-2: 5’ end—TGGCGGCCG*ctcgag*ATGACCTTGAAAATCGCATTTTTCA and 3’ end—ATCAAGCTC*gggccc*TTATTCAATATCAACAATCGGACTAT; EAT-18: 5’ end–TGGCGGCCG*ctcgag*ATGCGAAGCCTGGAGCGAAT and 3’ end—ATCAAGCTC*gggccc*TCAAAGTGTTGATCGCATTTCCTCA). For biochemistry and immunofluorescence assays, *Cel-*EAT-2 was tagged with GFP in between the transmembrane regions 3 and 4 between leucine 377 and 378; EAT-18 was tagged with the 6xHis tag at the C-terminal. Full-length sequences of *A*. *suum* EAT-2 (Accession number: GS_09411) and EAT-18 were amplified from total RNA extracted from the dissected whole pharynx of *A*. *suum*. Briefly, TRIzol Reagent (Life Technologies Inc., USA) was used to extract total RNA from *A*. *suum* adult worms. cDNA was synthesized by using SuperScript VILO Master Mix (Life Technologies Inc., USA) and served as a template for the amplification. Full-length product was sub-cloned into pTB207 expression vector by adding *XhoI* and *ApaI* restriction enzyme sites respectively to the forward primer (5’ end: TGGCGGCCG*ctcgag*ATGCAAATATTTTCTATGGTAATT) and reverse primer (3’ end: ATCAAGCTC*gggccc*TTAATTCCATACGTTTGGGG) using In-Fusion cloning. Z-competent *E*. *coli* JM109 cells (Zymo Research, USA) were used for the transformation of the ligated product. The final cloned constructs of all the plasmids were sequenced with pTB207 vector primers (forward, T7 and reverse, SP6). Only positive clones were used for cRNA synthesis using *in vitro* transcription with the mMessage mMachine T7 transcription kit (Life Technologies Inc., USA), and the cRNA was aliquoted and stored at -80°C.

### Electrophysiology

#### Two-electrode voltage-clamp in *Xenopus* oocytes

Oocyte injections and two-electrode electrophysiology recordings were performed as previously described [48].

#### *A*. *suum* current-clamp recordings from the pharynx

*A*. *suum* pharyngeal dissections and electrophysiology recordings were adapted from Martin [49]. Briefly, pharynx was dissected out from the head region of the worm and mounted on Sylgard (Sigma-Aldrich Inc., USA)—lined in a double jacketed bath chamber maintained at 28°C. The muscle layer surrounding the anterior 3^rd^ of the pharynx was preserved for anchoring. The intestine attached to the posterior end of the pharynx was used for stretching and pinning down. The preparation was continuously perfused with calcium-free *Ascaris* Perienteric Fluid-Ringer (calcium-free APF-Ringer) composition (mM): NaCl 23, Na-acetate 110, KCl 24, MgCl_2_ 11, glucose 11, and HEPES 5; NaOH or acetic acid was added to adjust the pH to 7.6. The experimental compounds were dissolved in calcium-free APF-Ringer and applied as described in the results. The rate of localized perfusion was 3.5–4 ml min^−1^ through a 20-gauge needle, which was placed directly above the recording region of the pharynx. The pharyngeal preparations with resting membrane potentials less than -15 mV and the resting conductances less than 250 μS were selected for analysis. We used 3 M potassium acetate in our micropipettes to get the final resistances of 4–7 MΩ for the voltage sensing and 0.5–1 MΩ for the current injecting electrode for current-clamp recordings. The current-injecting electrode injected hyperpolarizing step currents of −1000 nA for 500 ms at 0.3 Hz. Pharyngeal preparations with constant resting membrane potentials more negative than −15 mV for 20 min and a stable input conductance of <250 μS were selected for the recordings.

#### Data analysis

GraphPad Prism 8.0 software (GraphPad Software Inc., USA) was used to analyze the data. In two-electrode voltage-clamp recordings, the peak currents were measured and normalized to 100 μM ACh response and expressed as mean ± S.E.M. The data for sigmoid concentration-response curves were fitted to the Hill equation [17]. We used One-way analysis of variance (ANOVA) and Extra sum of squares F-test to test statistical differences (statistically different if *p*<0.05). Tukeys’ multiple comparison was used as a *post-hoc* test.

In current-clamp recordings, the peak changes in membrane conductance (δG_*max*_) in response to drug applications were normalized to δG response to ACh application (100 μM ACh, applied for 10s) within each preparation. We constructed the sigmoidal concentration-response plots by fitting the data by nonlinear regression to determine the *pEC*_*50*_ and the maximal response (R_max_). Extra sum of squares F-test was used to test statistical differences between *pEC*_*50*_, slope, and maximal response. The significance levels were set to *P*<0.05.

### Biochemistry

#### Immunostaining for confocal microscopy

Oocytes were prepared for confocal imaging following the previously published protocol [50]. In brief, oocytes were fixed in 4% paraformaldehyde at 4°C overnight. Fixed oocytes were embedded in 3% low-melting point agarose, and 50-μm thick slices were cut using a vibratome. The oocyte slices were blocked with 0.2% Bovine Serum Albumin (BSA) plus 0.1% Tween 20 in PBS overnight at 4°C. This was followed by incubation with the primary antibody (ABfinity Histidine tag recombinant rabbit oligoclonal antibody at 1:500 dilution for detecting *Cel-*EAT-18c, ThermoFisher#A-710286, and goat anti-GFP rabbit IgG antibody for detecting *Cel-*EAT-2, Abcam # ab6673, 1:1000) overnight at 4°C, and then incubated with a fluorescent secondary antibody (Alexa-Fluor 488 goat anti-rabbit IgG antibody, 1:1000, ThermoFisher#A-11008; Alexa-Fluor Plus 680 donkey anti-goat IgG secondary antibody, ThermoFisher#A-32860, 1:15000) for 1 hour at room temperature. The slices were mounted on glass slides using a Fluoromount-G mounting medium (Thermo Fisher Scientific, USA) followed by confocal imaging (Leica SP5 X MP confocal/multiphoton microscope system).

#### Western blot analysis using *Xenopus* oocytes

Oocyte protein extraction and Western blot analysis protocol were adapted from Lin-Moshier & Merchant [51] with the following modifications. Ten oocytes with currents ≥ 500 nA in response to 100 μM ACh were pooled and suspended in 100 μL of homogenization buffer (50 mM HEPES, pH = 7.5, 150 mM NaCl, 1 mM PMSF, 1 mM EDTA, pH = 8.0 and Protease Inhibitor cocktail, Sigma Aldrich, MO, USA). The homogenized sample was centrifuged at 800*g* for 5 min at 4°C, and the supernatant was transferred into a clean tube. The centrifugation step was repeated twice to ensure complete removal of the yolk particles. Samples were boiled with Laemmli buffer for 5 min and then subjected to electrophoresis (4–12% Bis-Tris gel for *Cel-*EAT-2-GFP and 16% Tricine gels for *Cel-*EAT-18-His). The gels were blotted onto PVDF membranes and blocked with BlockOut blocking buffer (Rockland Immunochemicals Inc., PA, USA). The blots were probed with a 1:10000 dilution of primary antibody (mouse monoclonal anti-GFP antibody to detect GFP tagged *Cel-*EAT-2, Proteintech # 66002-1-Ig; HRP-conjugated anti-His antibody to detect His tagged *Cel-*EAT-18, Proteintech # HRP-66005) at 4°C overnight. HRP conjugated anti-mouse antibody at 1:10000 dilution was used as the secondary antibody (SA00001-1) for GFP tagged protein. Immunoreactivity was visualized by enhanced chemiluminescence (GE healthcare, IL, USA).

#### Co-immunoprecipitation using *Xenopus oocytes* membrane extracts

*Xenopus laevis* oocytes were processed as described previously; anti-GFP-Trap-A beads (ChromoTek, Germany) were used for immunoprecipitation [22]. Laemmli buffer was used to recover the immunoprecipitates, and eluates were analyzed separately using the following primary antibodies: mouse monoclonal anti-GFP antibody (Proteintech # 66002-1-Ig, 1:10000), HRP-conjugated anti-His antibody (Proteintech # HRP-66005). HRP conjugated anti-mouse antibody was used as the secondary antibody (1:10000; SA00001-1). Chemiluminescent reagent (GE healthcare, IL, USA) was used for detection.

## Supporting information

S1 Fig Amino acid sequence alignment of *Cel*-EAT-2, *Asu*-EAT-2, and human-α7 nAChR subunits. The signal peptide (olive green), ACh-binding loops A–C (purple), cys-loop (orange), and transmembrane regions TM1–TM4 (light blue) are indicated. The vicinal cysteines (grey box) are absent in the C-binding loop of the EAT-2 protein. The conserved ligand binding residues of human-α7 subunits are highlighted in blue color in loops A-C and in maroon color in loops D-F. The residues not conserved in EAT-2 proteins are in grey boxes in the loops. The negatively charged acid residues flanking the transmembrane-2 region are highlighted in orange.
(TIFF)

S2 Fig Comparison of EAT-18 protein sequences and genomic organization. **(A)** Amino acid sequence alignment of *Asu*-EAT-18, *Cel*-EAT-18c, and *Cel*-EAT-18d. The predicted transmembrane domain is highlighted in blue. **(B)** Genomic organization of *lev-10* and *eat-18* (WormBase ParaSite). Purple boxes indicate coding regions; dark purple boxes represent 5’ and 3’ untranslated region of the transcript. The first exon of the *eat-18* is contained in the first intron of *lev-10*. The second exon of *eat-18 isoform c* is spliced to the second exon of lev-10 by using a different frame, which ends 16 bp after the splice site. The second exon of *eat-18 isoform c is* spliced to the third exon. **(C)** Predicted transmembrane topology of *Cel-*EAT-18c using Phobius.
(TIF)

S3 Fig Pharmacology of the *Cel-*EAT-2 nicotinic acetylcholine receptor expressed in *Xenopus* oocytes. **(A)** Representative traces of methacholine and butyrylcholine concentration-response relationships on the *Cel-*EAT-2 receptor. **(B)** Representative traces & acetylcholine concentration-response curves for *Cel*-EAT-2 receptor in the presence of 10 μM derquantel (der, n = 6) and 30 μM paraherquamide (para, n = 6). The *pEC*_*50*_ and *I*_*max*_ values (expressed as mean±SEM) were: 4.9±0.0 and 68.4±2.1% in the presence of derquantel; 4.7±0.1 and 40.2±2.7% in the presence of 30 μM paraherquamide. Both the antagonists did not produce a shift in *pEC*_*50*_ but reduced the efficacy of the acetylcholine on the *Cel-*EAT-2 receptor significantly (****P < 0.0001, Extra sum of squares F-test).
(TIF)

S4 Fig Representative traces of current-clamp recordings from the pharynx of *A*. *suum* showing the pharmacological effect of selected agonists. **(A)** Representative trace showing the conductance changes produced in response to the application of selected nicotinic agonists and cholinergic anthelmintics. **(B)** Representative trace showing concentration-dependent effects on the depolarization to the application of increasing concentrations of acetylcholine. **(C)** Representative trace showing concentration-dependent effects on the depolarization to the application of increasing concentrations of nicotine.
(TIF)

S5 Fig Representative traces of current-clamp recordings from the pharynx of *A*. *suum* showing the pharmacological effect of selected antagonists. The traces show a reduction in acetylcholine (10 and 100 μM) induced depolarizations in the presence of antagonists (30μM).
(TIF)

S6 Fig Pharmacological characterization of nAChRs expressed in the pharynx of *A*. *suum*. Functional profile of selected vertebrate nAChR antagonists (30μM) producing % inhibition of 100μM ACh membrane conductance (δG; expressed as mean ± SEM, %) in the *A*. *suum* pharynx: d-Tubocurarine (d-TC; 94.6±0.2) > mecamylamine (mec; 92.2±1.9) > methyllycaconitine (MLA; 62.6±3.7) > paraharquamide (para; 37.2±8.7) > derquantel (der; 30.6±7.0) > hexamethonium (hexa; 26.8±1.9) > dihydro-β-erythroidine (DHβE; 17.9±5.0).
(TIF)

S7 Fig Pharmacology of the *Asu-*EAT-2 nicotinic acetylcholine receptor expressed in *Xenopus* oocytes. **(A)** Current sizes (mean ± S.E.M) produced in response to 100 μM acetylcholine on *Asu-*EAT-2 nAChR. Black bar: *Asu*-EAT-2 + *Asu*-EAT-18 + *Asu*-RIC-3 (n = 11). Olive green bar: *Asu*-EAT-2 + *Asu*-EAT-18 + *Asu*-RIC-3 + *Asu*-UNC-50 + *Asu*-UNC-74 (n = 6). *Asu*-EAT-2 and *Asu*-EAT-18 did not form a functioning receptor on their own. Un-injected oocytes were used as a negative control. Black boxes indicate the presence of corresponding cRNA, and empty boxes indicate the absence of cRNA in the mix. **(B)** Representative traces of rank order series for nAChR agonists and anthelmintics on *Asu-*EAT-2 nAChR; nicotine (nic), cytisine (cyt), levamisole (lev), bephenium (bep), dimethylphenylpiperazinium (DMPP), oxantel (oxa), epibatidine (epi), choline (cho), thenium (the), tribendimidine (tri), pyrantel (pyr) morantel (mor). **(C)** Representative trace of acetylcholine concentration-response relationship for *Asu-*EAT-2 nAChR. **(D)** Representative trace showing inhibition of acetylcholine mediated currents by the selected antagonists (30 μM); d-tubocurarine (d-TC), dihydro-β-erythroidine (DhβE), mecamylamine (mec), methyllycaconitine (MLA), hexamethonium (hexa) and derquantel (der).
(TIF)

S8 Fig Localization of *Asu-eat-2* and *Asu-eat-18* mRNA in somatic muscle cells of the *A*. *suum* worm. Single-cell RT-PCR of *Asu-eat-2* (lanes 2, 6), *Asu-eat-18* (lanes 3, 7) and gapdh control (lanes 4,8) in somatic muscle cells (n = 10). Lane 1, FastRuler High Range DNA ladder; negative control- no-template controls for *Asu-eat-2* (lane-6), *Asu-eat-18* (lanes 7) and *gapdh* (lane-8).
(TIF)

S9 Fig Effect of different EAT-18 homologs on pharmacology of the *Cel-*EAT-2 receptor. **(A)** Representative trace and bar graph showing functional profile of agonists (100 μm; except tribendimidine, 30 μm) on *Cel-*EAT-2 + *Asu-*EAT-18 + *Asu-*RIC-3 mix; nicotine (nic), cytisine (cyt), levamisole (lev), bephenium (bep), tribendimidine (tri), pyrantel (pyr). **(B)** Concentration-response curves for acetylcholine application on *Cel-*EAT-2 + *Cel-*EAT-18c mix (black curve), *Cel-*EAT-2 + *Cel-*EAT-18c + *Asu-*RIC-3 (blue curve) and *Cel-*EAT-2 + *Asu-*EAT-18 + *Asu-*RIC-3 mix (green curve). **(C)** Bar graphs showing significant effect of using different EAT-18 proteins with *Cel-eat-2* on *pEC*_*50*_.^**^P < 0.01, ^***^P < 0.001; significantly different as indicated; based on Extra sum of squares F-test.
(TIF)

S10 Fig Uncropped Western blots. **(A)** Uncropped western blots corresponding to Fig 5E. **(B)** Uncropped western blots corresponding to Fig 5F. Dashed blue regions represent the cropped regions used in the main figures.
(TIF)

S11 Fig Protein sequence alignment of the EAT-2 subunit from multiple parasitic nematode species. The signal peptide (olive green), ACh-binding loops A–C (pink), loops D-F (green), cys-loop (grey), and transmembrane regions TM1–TM4 (blue) are indicated. The conserved ligand binding residues are highlighted in blue color in loops A-C and in maroon color in loops D-F.
(TIF)

S12 Fig Amino acid sequence alignment of EAT-18 from multiple parasitic nematode species. The transmembrane domain is highlighted in blue.
(TIF)

S1 Table Rank order potencies of nAChR agonists, antagonists, and cholinergic anthelmintics in *A*. *suum* pharyngeal nAChRs observed from our study, *A*. *suum* somatic muscle nAChRs and nAChRs of the vertebrate hosts. ACh (acetylcholine), nic (nicotine), cyt (cytisine), epi (epibaditine), DMPP (dimethylphenylpiperazine), chol (choline), pyr (pyrantel), oxa (oxantel), bep (bephenium), the (thenium), lev (levamisole), met (methyridine), d-TC (d-tubocurarine), mec (mecamylamine), MLA (methyllycaconitine), para (paraherquamide), der (derquantel), hexa (hexamethonium) and DHβE (Dihydro-β-erythroidine).
(DOCX)

S1 Data Effect of selected muscarinic agonists and antagonists on the *A*. *suum* pharynx. (DOCX)
