# Supplementary figures and images for: EAT-18 is an essential auxiliary protein interacting with the non-alpha nAChR subunit EAT-2 to form a functional receptor
Source: PLoS Pathog. 2020 Apr 3;16(4):e1008396. doi: 10.1371/journal.ppat.1008396 (PMC7173930; doi:10.1371/journal.ppat.1008396)

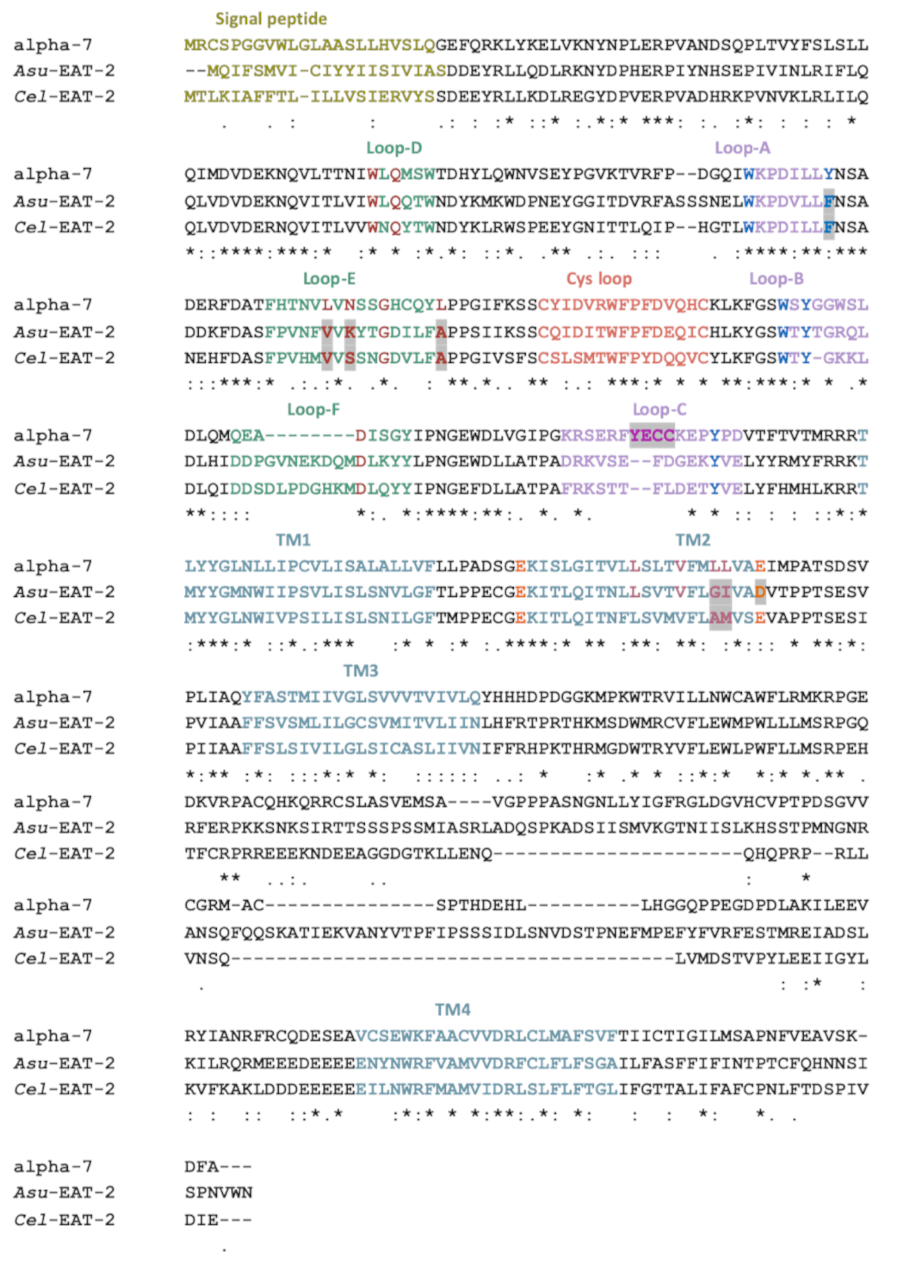

Supplement: S1 Fig — The signal peptide (olive green), ACh-binding loops A–C (purple), cys-loop (orange), and transmembrane regions TM1–TM4 (light blue) are indicated. The vicinal cysteines (grey box) are absent in the C-binding loop of the EAT-2 protein. The conserved ligand binding residues of human-α7 subunits are highlighted in blue color in loops A-C and in maroon color in loops D-F. The residues not conserved in EAT-2 proteins are in grey boxes in the loops. The negatively charged acid residues flanking the transmembrane-2 region are highlighted in orange. (TIFF) [file ppat.1008396.s001.tiff]

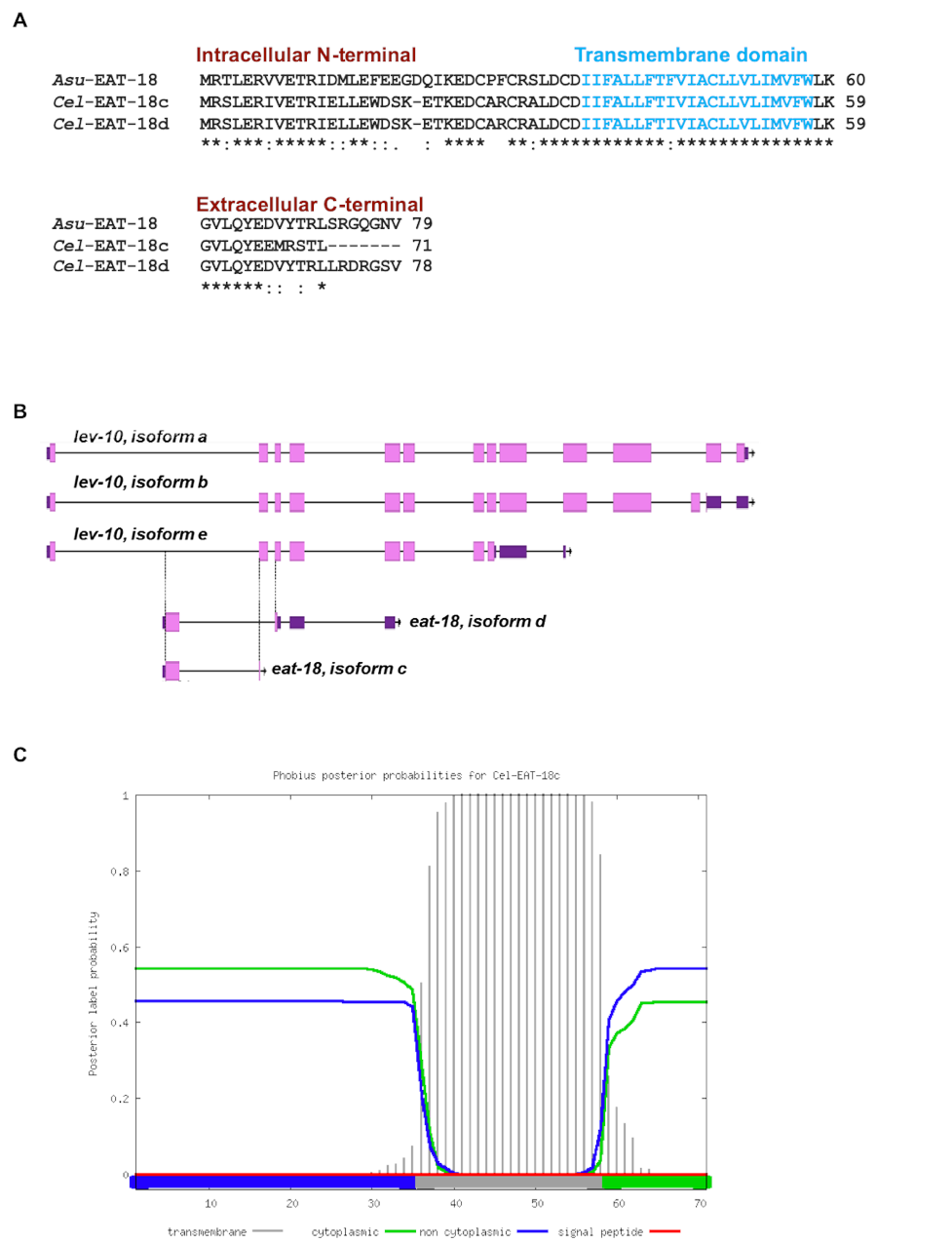

Supplement: S2 Fig — (A) Amino acid sequence alignment of Asu-EAT-18, Cel-EAT-18c, and Cel-EAT-18d. The predicted transmembrane domain is highlighted in blue. (B) Genomic organization of lev-10 and eat-18 (WormBase ParaSite). Purple boxes indicate coding regions; dark purple boxes represent 5’ and 3’ untranslated region of the transcript. The first exon of the eat-18 is contained in the first intron of lev-10. The second exon of eat-18 isoform c is spliced to the second exon of lev-10 by using a different frame, which ends 16 bp after the splice site. The second exon of eat-18 isoform c is spliced to the third exon. (C) Predicted transmembrane topology of Cel-EAT-18c using Phobius. (TIF) [file ppat.1008396.s002.tif]

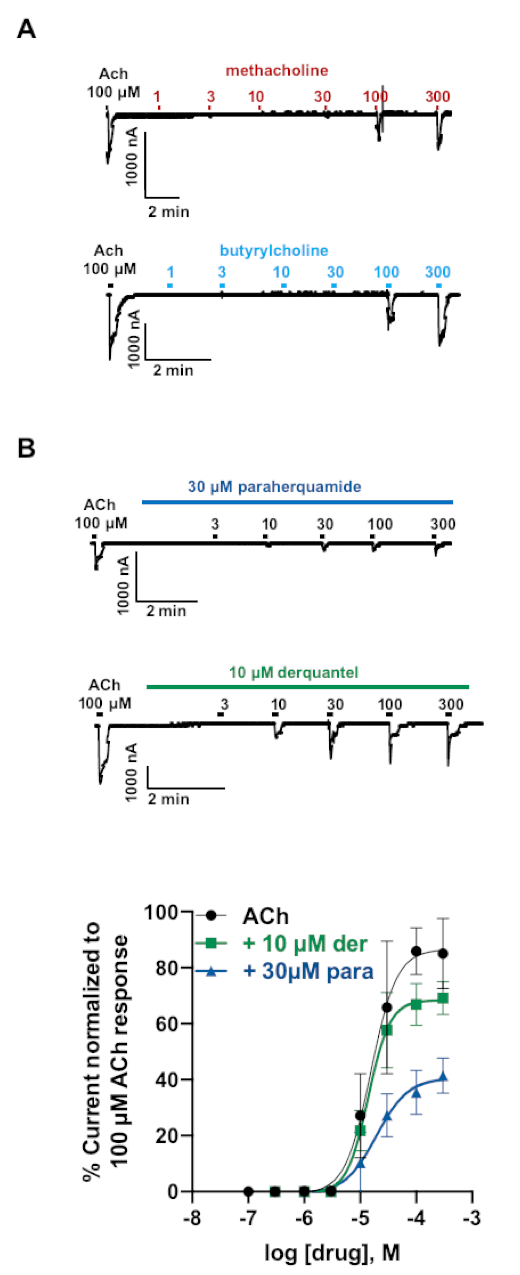

Supplement: S3 Fig — (A) Representative traces of methacholine and butyrylcholine concentration-response relationships on the Cel-EAT-2 receptor. (B) Representative traces & acetylcholine concentration-response curves for Cel-EAT-2 receptor in the presence of 10 μM derquantel (der, n = 6) and 30 μM paraherquamide (para, n = 6). The pEC50 and Imax values (expressed as mean±SEM) were: 4.9±0.0 and 68.4±2.1% in the presence of derquantel; 4.7±0.1 and 40.2±2.7% in the presence of 30 μM paraherquamide. Both the antagonists did not produce a shift in pEC50 but reduced the efficacy of the acetylcholine on the Cel-EAT-2 receptor significantly (****P < 0.0001, Extra sum of squares F-test). (TIF) [file ppat.1008396.s003.tif]

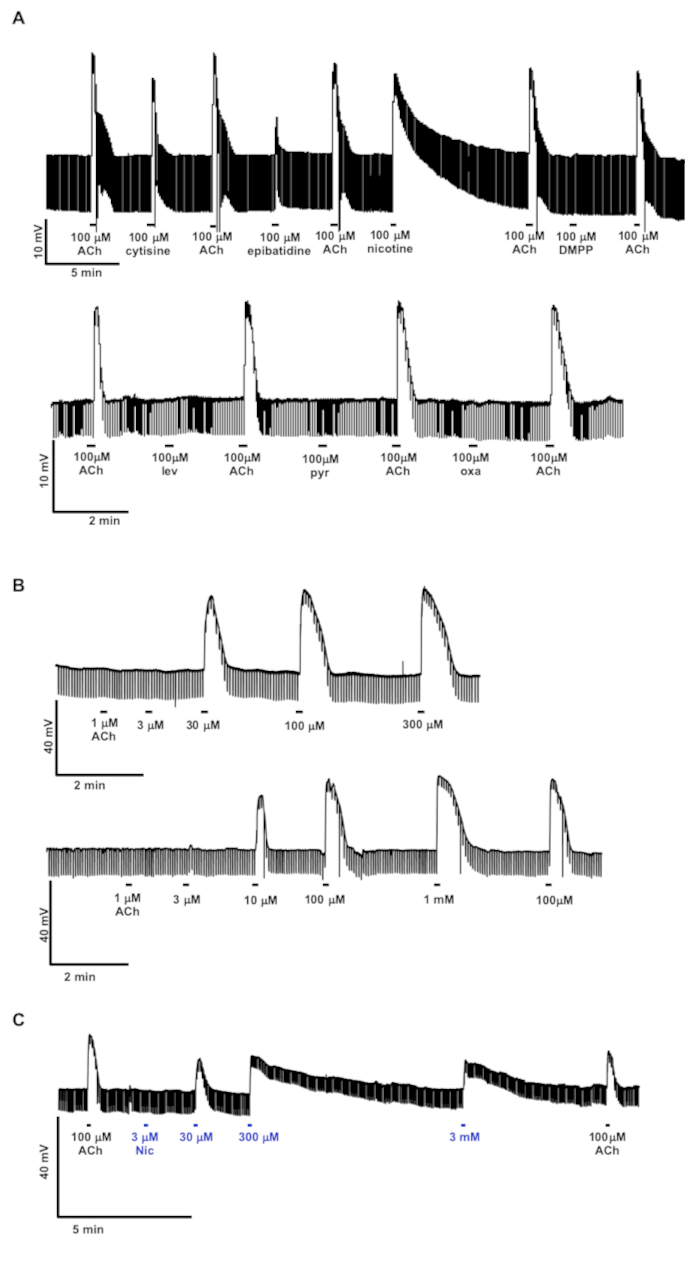

Supplement: S4 Fig — (A) Representative trace showing the conductance changes produced in response to the application of selected nicotinic agonists and cholinergic anthelmintics. (B) Representative trace showing concentration-dependent effects on the depolarization to the application of increasing concentrations of acetylcholine. (C) Representative trace showing concentration-dependent effects on the depolarization to the application of increasing concentrations of nicotine. (TIF) [file ppat.1008396.s004.tif]

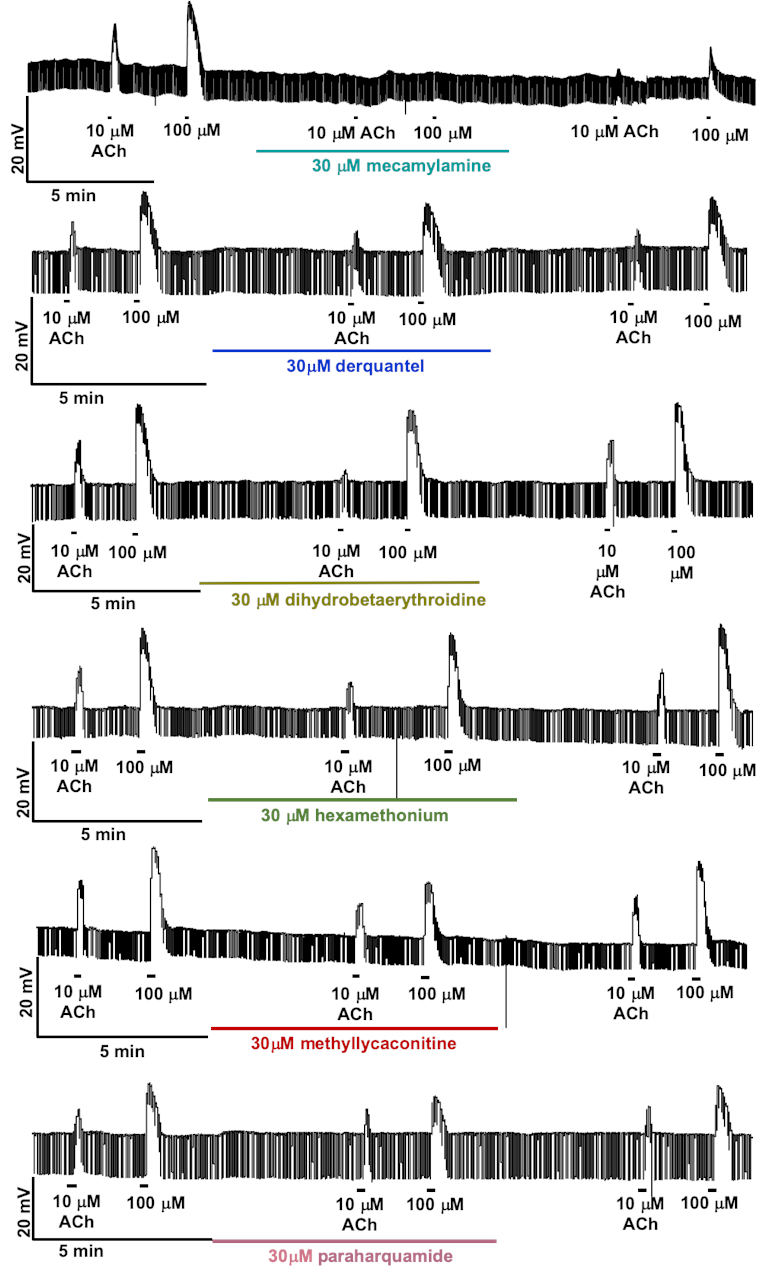

Supplement: S5 Fig — The traces show a reduction in acetylcholine (10 and 100 μM) induced depolarizations in the presence of antagonists (30μM). (TIF) [file ppat.1008396.s005.tif]

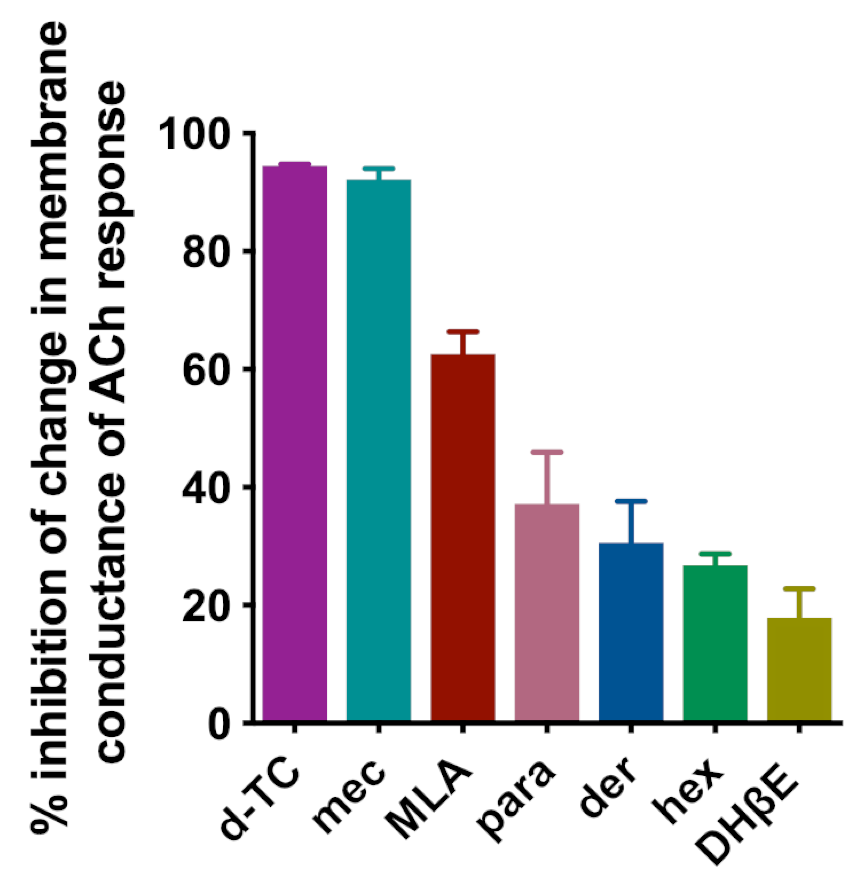

Supplement: S6 Fig — Functional profile of selected vertebrate nAChR antagonists (30μM) producing % inhibition of 100μM ACh membrane conductance (δG; expressed as mean ± SEM, %) in the A. suum pharynx: d-Tubocurarine (d-TC; 94.6±0.2) > mecamylamine (mec; 92.2±1.9) > methyllycaconitine (MLA; 62.6±3.7) > paraharquamide (para; 37.2±8.7) > derquantel (der; 30.6±7.0) > hexamethonium (hexa; 26.8±1.9) > dihydro-β-erythroidine (DHβE; 17.9±5.0). (TIF) [file ppat.1008396.s006.tif]

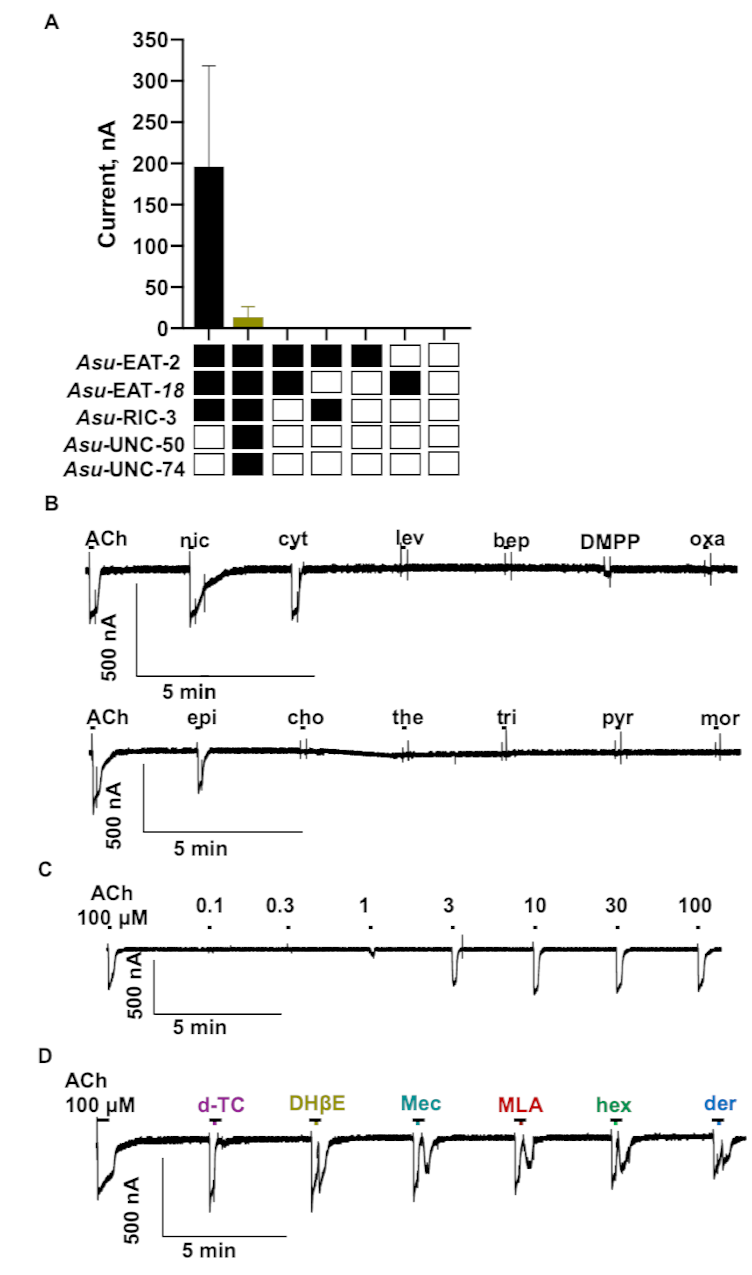

Supplement: S7 Fig — (A) Current sizes (mean ± S.E.M) produced in response to 100 μM acetylcholine on Asu-EAT-2 nAChR. Black bar: Asu-EAT-2 + Asu-EAT-18 + Asu-RIC-3 (n = 11). Olive green bar: Asu-EAT-2 + Asu-EAT-18 + Asu-RIC-3 + Asu-UNC-50 + Asu-UNC-74 (n = 6). Asu-EAT-2 and Asu-EAT-18 did not form a functioning receptor on their own. Un-injected oocytes were used as a negative control. Black boxes indicate the presence of corresponding cRNA, and empty boxes indicate the absence of cRNA in the mix. (B) Representative traces of rank order series for nAChR agonists and anthelmintics on Asu-EAT-2 nAChR; nicotine (nic), cytisine (cyt), levamisole (lev), bephenium (bep), dimethylphenylpiperazinium (DMPP), oxantel (oxa), epibatidine (epi), choline (cho), thenium (the), tribendimidine (tri), pyrantel (pyr) morantel (mor). (C) Representative trace of acetylcholine concentration-response relationship for Asu-EAT-2 nAChR. (D) Representative trace showing inhibition of acetylcholine mediated currents by the selected antagonists (30 μM); d-tubocurarine (d-TC), dihydro-β-erythroidine (DhβE), mecamylamine (mec), methyllycaconitine (MLA), hexamethonium (hexa) and derquantel (der). (TIF) [file ppat.1008396.s007.tif]

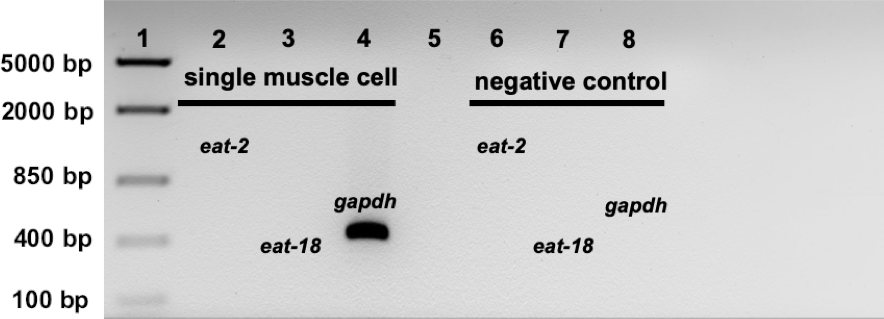

Supplement: S8 Fig — Single-cell RT-PCR of Asu-eat-2 (lanes 2, 6), Asu-eat-18 (lanes 3, 7) and gapdh control (lanes 4,8) in somatic muscle cells (n = 10). Lane 1, FastRuler High Range DNA ladder; negative control- no-template controls for Asu-eat-2 (lane-6), Asu-eat-18 (lanes 7) and gapdh (lane-8). (TIF) [file ppat.1008396.s008.tif]

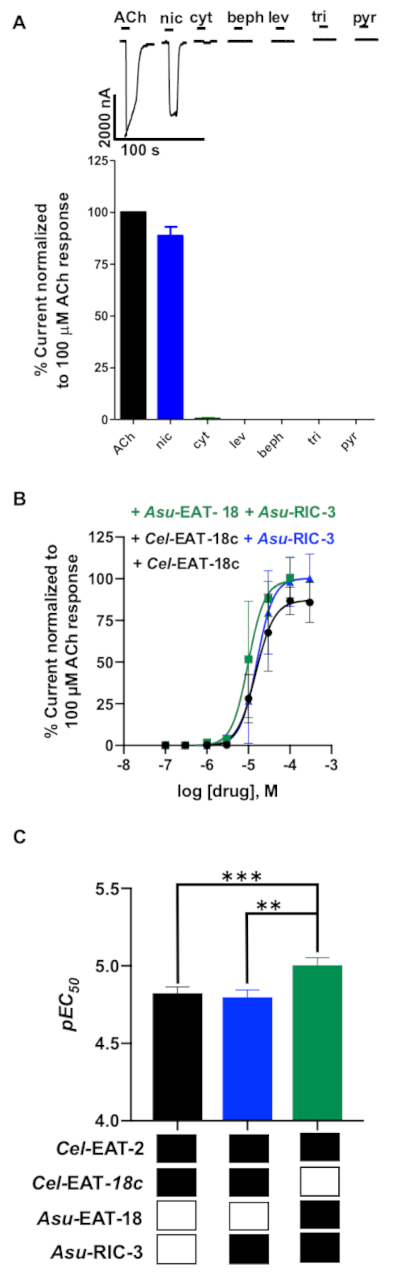

Supplement: S9 Fig — (A) Representative trace and bar graph showing functional profile of agonists (100 μm; except tribendimidine, 30 μm) on Cel-EAT-2 + Asu-EAT-18 + Asu-RIC-3 mix; nicotine (nic), cytisine (cyt), levamisole (lev), bephenium (bep), tribendimidine (tri), pyrantel (pyr). (B) Concentration-response curves for acetylcholine application on Cel-EAT-2 + Cel-EAT-18c mix (black curve), Cel-EAT-2 + Cel-EAT-18c + Asu-RIC-3 (blue curve) and Cel-EAT-2 + Asu-EAT-18 + Asu-RIC-3 mix (green curve). (C) Bar graphs showing significant effect of using different EAT-18 proteins with Cel-eat-2 on pEC50.**P < 0.01, ***P < 0.001; significantly different as indicated; based on Extra sum of squares F-test. (TIF) [file ppat.1008396.s009.tif]

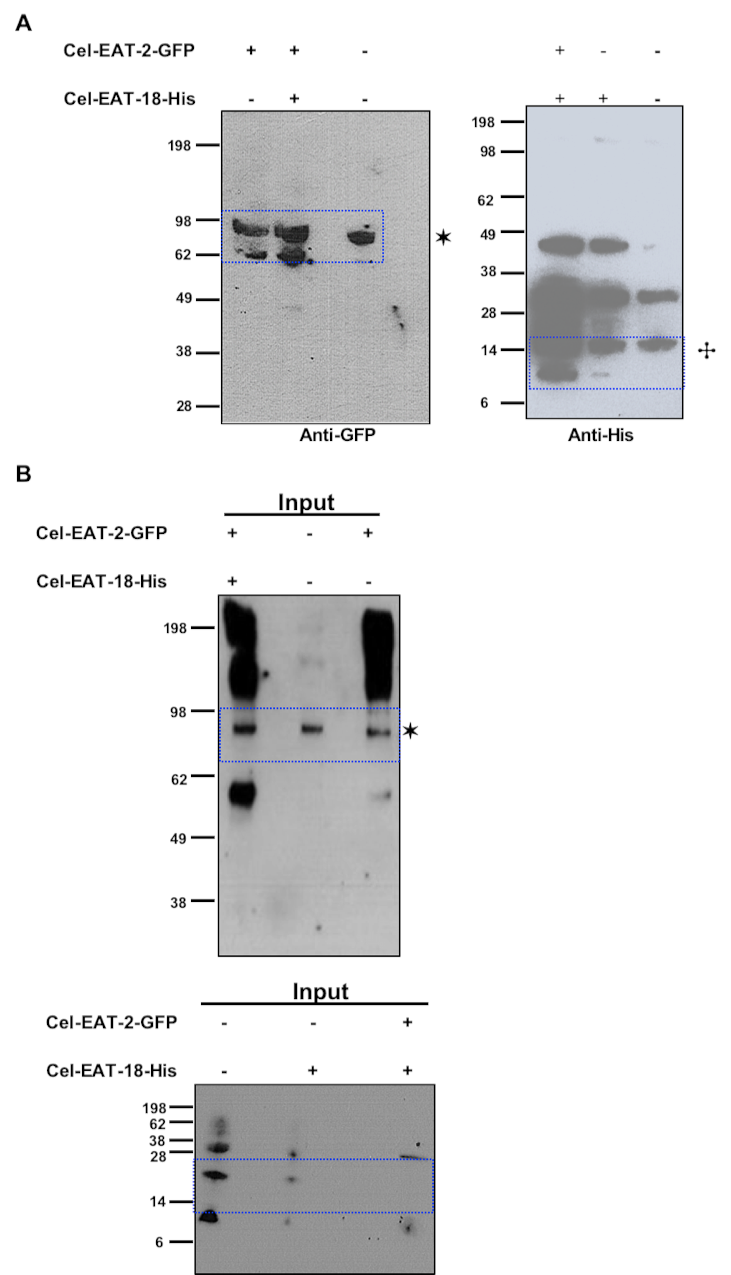

Supplement: S10 Fig — (A) Uncropped western blots corresponding to Fig 5E. (B) Uncropped western blots corresponding to Fig 5F. Dashed blue regions represent the cropped regions used in the main figures. (TIF) [file ppat.1008396.s010.tif]

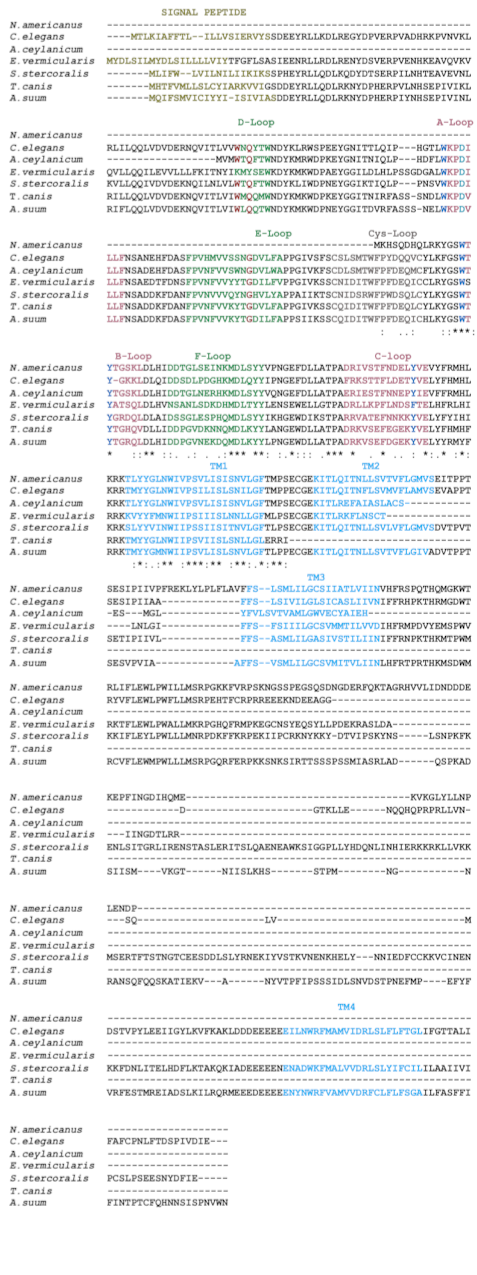

Supplement: S11 Fig — The signal peptide (olive green), ACh-binding loops A–C (pink), loops D-F (green), cys-loop (grey), and transmembrane regions TM1–TM4 (blue) are indicated. The conserved ligand binding residues are highlighted in blue color in loops A-C and in maroon color in loops D-F. (TIF) [file ppat.1008396.s011.tif]

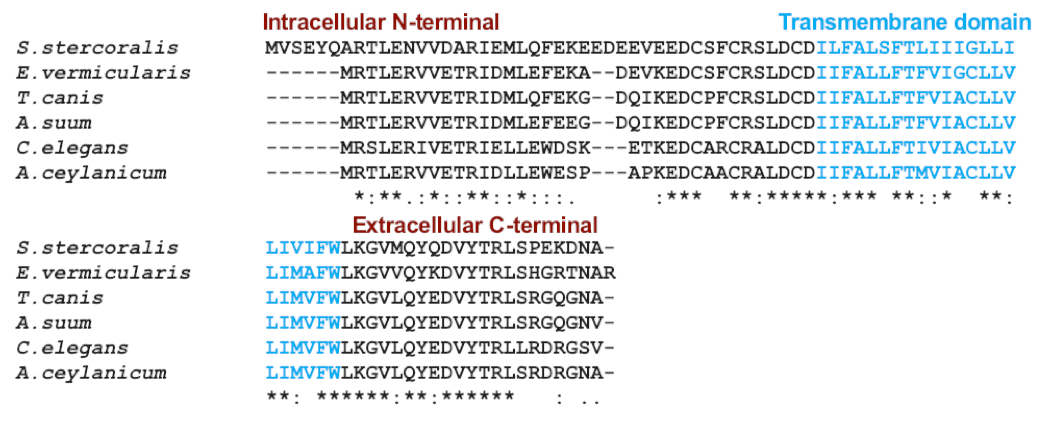

Supplement: S12 Fig — The transmembrane domain is highlighted in blue. (TIF) [file ppat.1008396.s012.tif]
