# Supplementary material for: EAT-18 is an essential auxiliary protein interacting with the non-alpha nAChR subunit EAT-2 to form a functional receptor
Source: PLoS Pathog. 2020 Apr 3;16(4):e1008396. doi: 10.1371/journal.ppat.1008396 (PMC7173930; doi:10.1371/journal.ppat.1008396)
Supplement: S1 Table — ACh (acetylcholine), nic (nicotine), cyt (cytisine), epi (epibaditine), DMPP (dimethylphenylpiperazine), chol (choline), pyr (pyrantel), oxa (oxantel), bep (bephenium), the (thenium), lev (levamisole), met (methyridine), d-TC (d-tubocurarine), mec (mecamylamine), MLA (methyllycaconitine), para (paraherquamide), der (derquantel), hexa (hexamethonium) and DHβE (Dihydro-β-erythroidine). (DOCX) [file ppat.1008396.s013.docx]

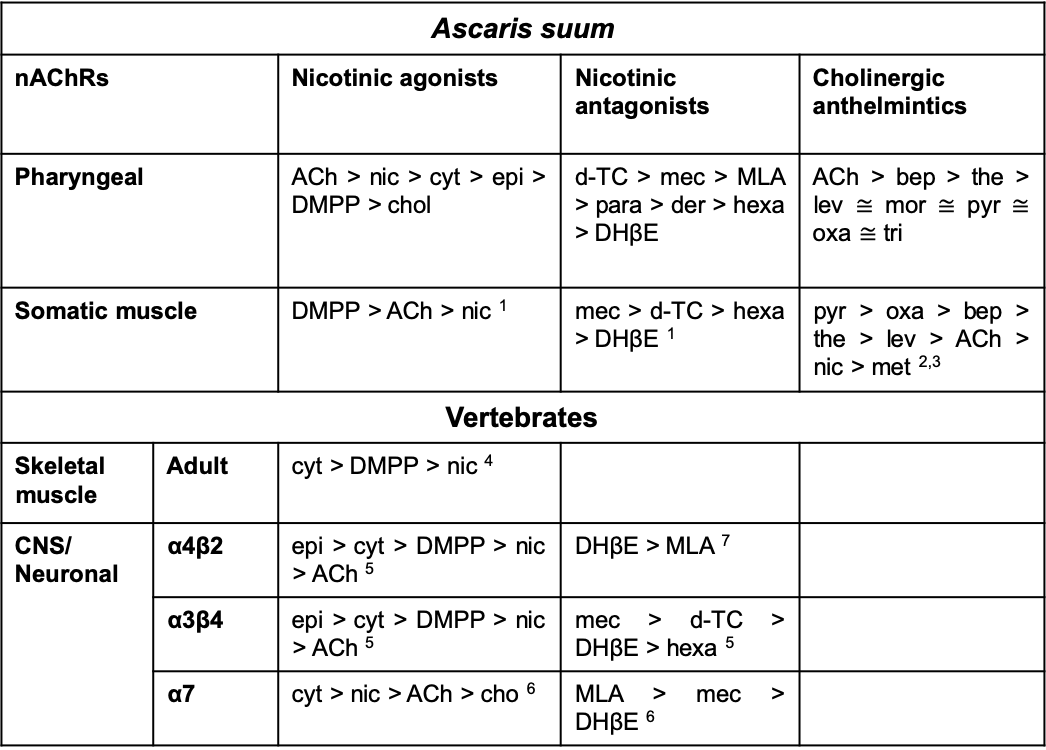


^1^ Colquhoun, L., Holden-Dye, L. & Walker, R.J. *J Exp Biol* **158**, 509-530 (1991).

^2^ Martin, R.J., Clark, C.L., Trailovic, S.M. & Robertson, A.P. *Int J Parasitol* **34**, 1083-1090 (2004).

^3^ Trailovic, S.M., Verma, S., Clark, C.L., Robertson, A.P. & Martin, R.J. *Int J Parasitol* **38**, 945-957 (2008).

^4^ Yost, C.S. & Winegar, B.D. *Cell Mol Neurobiol* **17**, 35-50 (1997) .

^5^ Wonnacot, S. & Barik, J. *Tocris Bioscience scientific review series* **28,**1-20 (2007).

^6^ Virginio, C., Giacometti, A., Aldegheri, L., Rimland, J.M. & Terstappen, G.C. Eur J Pharmacol **445**, 153-161 (2002).

^1^ Colquhoun, L., Holden-Dye, L. & Walker, R.J. *J Exp Biol* **158**, 509-530 (1991).

^2^ Martin, R.J., Clark, C.L., Trailovic, S.M. & Robertson, A.P. *Int J Parasitol* **34**, 1083-1090 (2004).

^3^ Trailovic, S.M., Verma, S., Clark, C.L., Robertson, A.P. & Martin, R.J. *Int J Parasitol* **38**, 945-957 (2008).

^4^ Yost, C.S. & Winegar, B.D. *Cell Mol Neurobiol* **17**, 35-50 (1997) .

^5^ Wonnacot, S. & Barik, J. *Tocris Bioscience scientific review series* **28,**1-20 (2007).

^6^ Virginio, C., Giacometti, A., Aldegheri, L., Rimland, J.M. & Terstappen, G.C. Eur J Pharmacol **445**, 153-161 (2002).
