# Supplementary material for: EAT-18 is an essential auxiliary protein interacting with the non-alpha nAChR subunit EAT-2 to form a functional receptor
Source: PLoS Pathog. 2020 Apr 3;16(4):e1008396. doi: 10.1371/journal.ppat.1008396 (PMC7173930; doi:10.1371/journal.ppat.1008396)
Supplement: S1 Data — (DOCX) [file ppat.1008396.s014.docx]

**S1 Data. Effect of selected muscarinic agonists and antagonists on the *A. suum* pharynx.**

Acetylcholine is a known agonist at nicotinic and muscarinic receptors in vertebrates. The rank order series of muscarinic agonists (all used at 100µM) observed for an *A. suum* muscarinic receptor expressed in yeast was ACh (100%) > carbachol (80%) ≅ arecoline (76%) > oxotremorine (61%) ≅ bethanechol (58%) > pilocarpine (14%)^1^. Here we tested the hypothesis that the observed change in membrane conductance response (δG) to ACh on the pharynx is produced by activation of nAChRs and mAChRs (GARs). Our pharyngeal preparations in this group had a resting membrane potential (RMP) of -17.6±1.3 mV and a resting conductance of 147.8±13.1 µS (n=8, mean±SE). We tested responses to the muscarinic agonists ACh, 5-methylfurmethiodide (MFI), oxotremorine, arecoline, pilocarpine (all 100µM, 10s application). In applications containing arecoline and pilocarpine, we used mecamylamine, a nAChR antagonist (30µM) to inhibit nAChRs and to allow only mAChR activation. A control application of ACh (100 µM, 10s) in the presence of mecamylamine was used for comparison of response to nAChR activation. We normalized the conductance change (δG) produced by the control ACh application (100 µM, 10s) to 100% in order compare the relative responses to other muscarinic agonists. The normalized responses to muscarinic agonists (mean ± SE, %, n=4) were MFI (4.0±0.6), oxotremorine (1.8±0.8), arecoline (2.6±2.0), pilocarpine (0.7±0.7). Mecamylamine inhibited 92% of the ACh δG response suggesting nicotinic receptor activation rather than the muscarinic receptor activation was responsible for the observed changes. Our results demonstrate the contribution of mAChRs to the acetylcholine induced conductance changes is negligible. Therefore, it was unnecessary to further use muscarinic receptor antagonists in our experiments to characterize the nAChRs. Other work has documented the phenomenon of concentration dependent reversible channel block produced by atropine (IC50 = 4-10µM) on α3β4, α3β2, α4β4 and α4β2 vertebrate nAChRs^2^.

^1^ Kimber M.J. et al. *Int J Parasitol* 39: 1215-1222 (2009).

^2^ Parker J.C., Sarkar D., Quick M.W. & Lester R.A. *Br J Pharmacol* 138: 801-810 (2003).
